# Phosphorylation and *O*-GlcNAcylation of the PHF-1 Epitope of Tau Protein Induce Local Conformational Changes of the C-Terminus and Modulate Tau Self-Assembly Into Fibrillar Aggregates

**DOI:** 10.3389/fnmol.2021.661368

**Published:** 2021-06-17

**Authors:** François-Xavier Cantrelle, Anne Loyens, Xavier Trivelli, Oliver Reimann, Clément Despres, Neha S. Gandhi, Christian P. R. Hackenberger, Isabelle Landrieu, Caroline Smet-Nocca

**Affiliations:** ^1^Risk Factors and Molecular Determinants of Aging-Related Diseases, U1167, Institut Pasteur de Lille, CHU Lille, INSERM, University of Lille, Lille, France; ^2^CNRS, ERL9002 – Integrative Structural Biology, Lille, France; ^3^Centre de Recherche Jean-Pierre AUBERT Neurosciences et Cancer, U1172, CHU Lille, INSERM, University of Lille, Lille, France; ^4^Université de Lille, CNRS, INRAE, Centrale Lille, Université d’Artois, Lille, France; ^5^Leibniz-Forschungsinstitut für Molekulare Pharmakologie, Berlin, Germany; ^6^Institut für Chemie, Humboldt-Universität zu Berlin, Berlin, Germany; ^7^Centre for Genomics and Personalised Health, Cancer and Ageing Research Program, School of Chemistry and Physics, Faculty of Science and Engineering, Institute of Health and Biomedical Innovation, Queensland University of Technology, Brisbane, QLD, Australia

**Keywords:** Alzheimer’s disease, microtubule-associated protein tau, phosphorylation, *O*-GlcNAc glycosylation, protein aggregation, NMR spectroscopy

## Abstract

Phosphorylation of the neuronal microtubule-associated Tau protein plays a critical role in the aggregation process leading to the formation of insoluble intraneuronal fibrils within Alzheimer’s disease (AD) brains. In recent years, other posttranslational modifications (PTMs) have been highlighted in the regulation of Tau (dys)functions. Among these PTMs, the *O*-β-linked N-acetylglucosaminylation (*O*-GlcNAcylation) modulates Tau phosphorylation and aggregation. We here focus on the role of the PHF-1 phospho-epitope of Tau C-terminal domain that is hyperphosphorylated in AD (at pS396/pS404) and encompasses S400 as the major *O*-GlcNAc site of Tau while two additional *O*-GlcNAc sites were found in the extreme C-terminus at S412 and S413. Using high resolution NMR spectroscopy, we showed that the *O*-GlcNAc glycosylation reduces phosphorylation of PHF-1 epitope by GSK3β alone or after priming by CDK2/cyclin A. Furthermore, investigations of the impact of PTMs on local conformation performed in small peptides highlight the role of S404 phosphorylation in inducing helical propensity in the region downstream pS404 that is exacerbated by other phosphorylations of PHF-1 epitope at S396 and S400, or *O*-GlcNAcylation of S400. Finally, the role of phosphorylation and *O*-GlcNAcylation of PHF-1 epitope was probed in *in-vitro* fibrillization assays in which *O*-GlcNAcylation slows down the rate of fibrillar assembly while GSK3β phosphorylation stimulates aggregation counteracting the effect of glycosylation.

## Introduction

Alzheimer’s disease (AD) is defined by two types of lesions, the extraneuronal senile plaques made of Aβ peptides and neurofibrillary degeneration constituted by intraneuronal inclusions of hyperphosphorylated Tau proteins. Tau is a microtubule (MT)-associated protein which is mainly expressed in neuronal axons under six alternatively spliced isoforms in adult brain. Although it has been shown that its primary function is promoting assembly and stability of MTs (Weingarten et al., 1975; Cleveland et al., 1977), other important functions have been described more recently at the cell membrane (Morris et al., 2011) and in the nucleus (Sultan et al., 2011; Mansuroglu et al., 2016) regulating cell signaling, genome expression and stability, and neuronal plasticity at synapses. The various functions of Tau are regulated by phosphorylation that was also shown to play a crucial role in Tau pathogenesis related to fibrillar aggregation in neuronal disorders referred to as tauopathies. In addition to phosphorylation, Tau is highly regulated in its physiological state by an intricate array of posttranslational modifications (PTMs) including ubiquitination, acetylation, N-glycosylation, and *O*-β-linked N-acetylglucosaminylation (*O*-GlcNAcylation) (Hanger et al., 1998; Morris et al., 2015).

**FIGURE 1:**
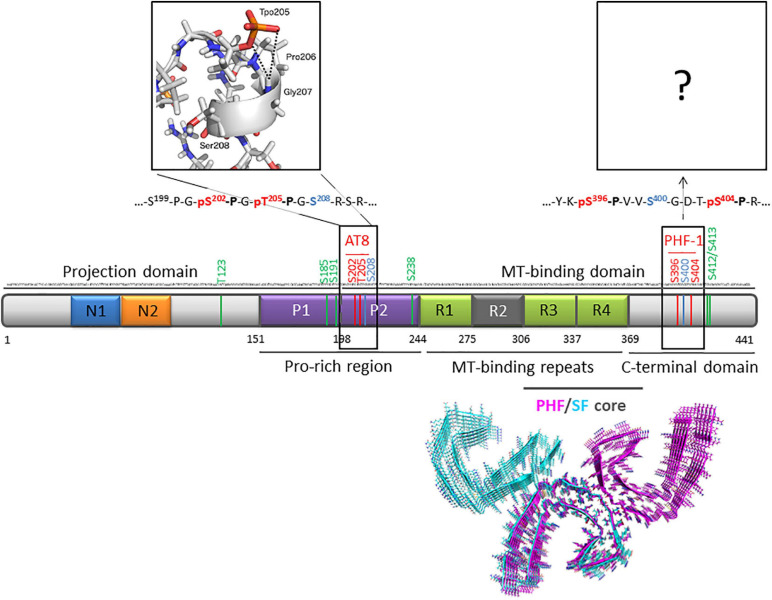
Primary structure of human Tau protein (2N4R isoform of 441 residues) indicating AT8 (pS202/pT205) and PHF-1 (pS396/pS404) phospho-epitopes (red) and *O*-GlcNAc sites (green). Sites that can be either phosphorylated or *O*-GlcNAcylated are indicated in blue. The inset depicts a representative conformation of AT8 phospho-epitope in its di-phosphorylated form obtained by NMR and molecular dynamics (Gandhi et al., 2015). Ultrastructure of PHF (PDB ID: 5O3L) and SF (PDB ID: 5O3T) cores of Tau protein from AD brain (tau 306-378) are depicted by pink and cyan ribbons, respectively (Fitzpatrick et al., 2017).

Tau is an intrinsically disordered protein and its primary structure is divided into four regions: an N-terminal projection domain, a proline-rich domain (PRD), a microtubule-binding domain (MTBD), and a C-terminal region (Figure 1) (Mandelkow et al., 1996; Buée et al., 2000). In its fibrillar state, however, Tau MTBD forms highly ordered structures that are disease-specific (Fitzpatrick et al., 2017; Falcon et al., 2018a,b; Fyfe, 2018). In AD, two types of filaments, the paired helical filaments (PHFs) and straight filaments (SFs), are the main constituents of the neurofibrillary inclusions consisting of the same protofilament structure, yet differing by the protofilament assembly (Fitzpatrick et al., 2017). In these structures, the region encompassing residues 306–378 of Tau MTBD adopt a cross-β/β-helix fold (Figure 1). While Tau is hyperphosphorylated in its disease-associated fibrillar state, phosphorylation sites (as well as other PTMs) were not observed in former cryo-EM structures of PHF-Tau from AD brains presumably due to PTM heterogeneity failing to provide a detail view of phosphate accommodation into filament assembly (Fitzpatrick et al., 2017). Tau filaments in AD and cortico-basal degeneration (CBD) were shown to be extensively modified by phosphorylation, acetylation, trimethylation and ubiquitination with acetylation and ubiquitination occurring mainly within the fibril cores (Arakhamia et al., 2020). In contrast, the large majority of phosphorylation sites are found in the proline-rich region and the C-terminal domain localized at the N- and C-terminal ends of MTBD, respectively (Hanger et al., 1992, 1998, 2007). It has been proposed that ubiquitin or poly-ubiquitin could stabilize the stacking of β-strands along the fibril axis and mediate inter-protofilament interfaces in straight filaments from AD (Arakhamia et al., 2020). Therefore, diverse PTM patterns could be crucial in mediating distinct packing of protofilaments leading to the ultra-structure polymorphism. However, despites increasing knowledge in site-specific characterization of Tau PTMs and structures of Tau filaments from various tauopathies, the mechanisms underlying fibrillization as well as the role of abnormal phosphorylation are still not fully understood. Besides detaching Tau from microtubules, abnormal phosphorylation was thought to alter Tau conformation inducing accumulation of free Tau in the cytosol into a pathological conformation prone to self-assembly that may convert normal Tau into abnormal oligomers that are not only the primary state of insoluble fibrils but also acting as seeds to propagate Tau pathology from neurons to neurons (Goedert et al., 2010, 2017).

The *O*-GlcNAc glycosylation is another dynamic PTM involved in regulation of Tau, notably through impairment of its phosphorylation and toxicity (Arnold et al., 1996; Liu et al., 2004, 2009; Yuzwa et al., 2008). While phosphorylation is dynamically regulated by hundreds of kinases and phosphatases, *O*-GlcNAcylation is only regulated by two antagonist enzymes, *O*-GlcNAc transferase (OGT) and *O*-GlcNAc hydrolase (OGA) (Hart, 1997, 2014; Hart et al., 2007; Bullen et al., 2014). It has been shown that *O*-GlcNAcylation of Tau does not alter microtubule polymerization but displays intrinsic properties to reduce *in vitro* fibrillization of Tau induced by heparin without altering its conformation (Yuzwa et al., 2012, 2014). It could rather act by increasing monomer solubility or directly hindering interactions at play in Tau self-assembly during the fibrillization process. Pharmacological increase of *O-*GlcNAcylation upon treatment of cells or mice with inhibitors of OGA leads to neuroprotective effects therefore constituting a potent strategy to tackle neurodegenerative diseases and providing a valuable alternative to kinase inhibitors (Yuzwa et al., 2012; Graham et al., 2014).

We have recently highlighted using high resolution NMR spectroscopy that S400 *O*-GlcNAcylation and S404 phosphorylation act as a unique motif regulated by a direct PTM crosstalk inducing each other chemical shift changes that suggest a spatial proximity between both sites in full-length Tau protein (Bourré et al., 2018; Theillet et al., 2012). However, the overall effect of *O*-GlcNAcylation in modulation of Tau phosphorylation is at least scarce in the context of a low phosphorylation level that mimics physiological phosphorylation state of Tau and absent in a context of high phosphorylation level mimicking the hyperphosphorylated state (Bourré et al., 2018). We have previously shown in peptides, however, that S400 *O*-GlcNAcylation could act as a direct mechanism that blocks the formation of the pathological PHF-1 phospho-epitope (pS396/pS400/pS404) in the C-terminal domain of Tau by disrupting the sequential phosphorylation by GSK3β (Smet-Nocca et al., 2011). Although it was shown that *O*-GlcNAcylation does not perturb the conformation of non-phosphorylated Tau (Yuzwa et al., 2014), another potential role relies on conformational changes occurring in (hyper)phosphorylated Tau in which *O*-GlcNAcylation could prevent abnormal conformations that promote self-assembly into insoluble filaments.

This points to a role of the *O*-GlcNAc modification in the direct crosstalk with GSK3β-mediated phosphorylation towards regulating Tau conformation and properties in fibrillar self-assembly. GSK3β is a constitutively active serine/threonine kinase involved in physiological Tau phosphorylation that regulates its binding to MT (Spittaels et al., 2000; Sun et al., 2002). Several studies have highlighted that GSK3β preferentially phosphorylates most of its substrates, including Tau, after prephosphorylation (priming) by other kinases such as Protein Kinase A, Casein Kinases 1 and 2, cdk5 or members of the mitogen-activated protein kinase family (Singh et al., 1995; Cho and Johnson, 2003; Li et al., 2006). In most cases, GSK3β phosphorylates (S/T)-XXX-(pS/pT) motifs (where pS/pT is the priming site) that is at a Ser/Thr residue located four residues N-terminal to the priming site, more rarely at a five or six residue N-terminal to the priming site (Leroy et al., 2010). This points towards a regulatory role of the *O*-GlcNAc modification either at the priming sites or GSK3β targeted sites. Interestingly, primed or unprimed Tau has a differential impact on its phosphorylation by GSK3β with functional modulation on MT binding (Cho and Johnson, 2003; Li et al., 2006). Tau encompasses as many as 24 strict consensus sequences for GSK3β, fourteen of these Ser/Thr pairs were found to be phosphorylated by GSK3β *in vitro* (Reynolds et al., 2002). Furthermore, it has been shown that phosphorylation of the PHF-1 epitope does not required priming by another kinase. Instead, priming can be performed by GSK3β itself in which phosphorylation of the S404 proline-directed site plays a critical role (Leroy et al., 2010). GSK3β plays also a key role in AD pathogenesis in which it contributes to several phosphorylation sites found in hyperphosphorylated PHF-Tau (Hanger et al., 1992; Morishima-Kawashima et al., 1995; Reynolds et al., 2002; Avila et al., 2010; Hernandez et al., 2012; Llorens-Maritin et al., 2014). In AD, it has been proposed that GSK3β could be the link between amyloid peptide and Tau phosphorylation since activation of GSK3β is regulated by signaling pathways targeted by amyloid peptide (Alvarez et al., 1999; Hernández et al., 2010). On the other hand, GSK3β regulates Aβ level and toxicity through Tau phosphorylation and neurodegeneration. Overexpression of GSK3β *in vivo* accelerates Tau-induced neurodegeneration while Tau-knockout mice did not exhibit deleterious effects of GSK3β overexpression indicating that Tau mediates GSK3β toxicity. Inhibitors of GSK3β reduce tauopathy and alleviate neurodegeneration *in vivo* (Noble et al., 2005), and are therefore considered as therapeutic agents (Engel et al., 2008; Hernandez et al., 2009; Medina et al., 2011) highlighting the role of GSK3β in Tau pathology.

In this study, we have investigated by NMR spectroscopy the phosphorylation pattern induced by GSK3β with or without priming by CDK2/cyclin A and its modulation by the direct crosstalk with *O*-GlcNAcylation. A per-residue resolution allowed a precise characterization of Tau PTMs at the protein level prior to investigating their role in Tau fibrillization *in vitro*. Furthermore, conformational changes induced by PTM combinations of Tau PHF-1 epitope was studied in chemically synthesized and modified peptides (Reimann et al., 2015, 2017; Schwagerus et al., 2016) by NMR chemical shift analyses.

## Materials and Methods

### Tau Mutants

TauS262A mutant was obtained by site-directed mutagenesis of Tau2N4R (longest isoform of 441 residues) (Despres et al., 2017). TauPHF1 mutant was obtained by gene synthesis with codon optimization for production in *E. coli*. Tau proteins were cloned into pET15b vector (Novagen) into NcoI/XhoI allowing removal of the sequence coding for the polyhistidine tag. TauPHF1 protein corresponds to a mutated form of Tau2N4R in which every Pro-directed Ser/Thr phosphorylation site as well as S262, S356 and S191 were mutated into Ala to prevent phosphorylation except those of the PHF-1 phospho-epitope (pS396/pS404).

### Production of Tau Proteins

TauS262A and TauPHF1 mutants were produced as described previously for wild-type Tau (Qi et al., 2017). Briefly, BL21(DE3) *E. coli* strains transformed with Tau mutants were grown in M9 minimal medium enriched with ^15^N as nitrogen source for ^15^N-labeling (6g Na_2_HPO_4_, 3g KH_2_PO_4_, 0.5g NaCl, 4g glucose, 1g ^15^NH_4_Cl, 0.5g ^15^N-Isogro^®^ (Sigma), 1 mM MgSO_4_, 10 ml MEM vitamin cocktails (Sigma), 100 mg ampicillin per liter) at 37°C until OD at 600 nm reached 0.8–0.9. For ^15^N/^13^C-labeling, 0.5g ^15^N/^13^C-Isogro^®^ (Sigma) and 2g ^13^C_6_-glucose instead of glucose were used. Then, cultures were induced by 0.5 mM isopropyl-thiogalactoside (IPTG) and cells were grown at 37°C for approximately 3 hours. The bacterial pellets harvested by centrifugation were resuspended in 40ml per liter of culture of cation exchange equilibrium buffer (50 mM Na_2_HPO_4_/NaH_2_PO_4_ pH 6.6, 2 mM EDTA) supplemented with 0.5% Triton X100, 2 mM DTT and cOmplete^TM^ EDTA-free protease inhibitor cocktail (Roche). Cell lysis was performed by high pressure homogenization and the soluble extract was obtained by centrifugation at 30,000 × *g* for 30 min at 4°C. Then, heating of the soluble extract at 80°C for 15 minutes followed by centrifugation was performed as a first purification step before cation-exchange chromatography (HiTrap SP 5 ml column, GE Healthcare). Homogeneous fractions containing full-length Tau proteins as checked by SDS-PAGE and MALDI-TOF mass spectrometry were pooled and buffer-exchanged in 50 mM ammonium bicarbonate prior to lyophilization.

### *O-*GlcNAcylation of Tau by OGT

ncOGT (110 kDa, nucleocytoplasmic isoform) and *O*-GlcNAc Tau (hereafter named Tau-G, Table 1) was obtained as described in Bourré et al. (2018). Briefly, 10 mg of lyophilized Tau protein was incubated at 800 μM with ncOGT at 0.5 mg/ml and 10 mM UDP-GlcNAc in OGT reaction buffer (50 mM KH_2_PO4/K_2_HPO4 pH 7.6, 150 mM NaCl, 1 mM EDTA, 0.5 mM THP, 12.5 mM MgCl_2_) at 31°C for 2 days. *O*-GlcNAcylated Tau protein was enriched by reverse phase high-pressure liquid chromatography (Zorbax 300SB-C8 column 5μm, Agilent). To increase amounts of Tau-G for NMR analyses, a second round of *O*-GlcNAcylation/purification was performed with the non-*O*-GlcNAc enriched fraction. Both *O*-GlcNAc-enriched fractions were pooled together. Following this procedure, an average of 1.8 *O*-GlcNAc per Tau molecule were found by MALDI-TOF mass spectrometry considering a mass increment of +203 Da per GlcNAc group (see Supplementary Figures 1E–G).

**TABLE 1:** Abbreviations used for the diverse PTM combination in Tau proteins in this study.

**Protein name**	**Posttranslational Modifications**
Tau	–
Tau-P	CDK2/cyclin A3 phosphorylation
Tau-PP	CDK2/cyclin A3 followed by GSK3β phosphorylation
Tau-P(GSK3)	GSK3β phosphorylation
Tau-G	OGT *O*-GlcNAcylation
Tau-G/P	OGT *O*-GlcNAcylation and CDK2/cyclin A3 phosphorylation
Tau-G/PP	OGT *O*-GlcNAcylation and CDK2/cyclin A3 followed by GSK3β phosphorylation
Tau-G/P(GSK3)	OGT *O*-GlcNAcylation and GSK3β phosphorylation

*Tau protein refers to either TauS262A or TauPHF1 mutants.*

### Phosphorylation of Tau by CDK2/Cyclin A

CDK2 phosphorylated at T160 was produced in *E. coli* by coexpression of human GST–CDK2 and *S. cerevisiae* GST–Cak1 using a pGEX vector (GE Healthcare), as described (Brown et al., 1999; Welburn and Endicott, 2005). Human cyclin A3 (residues 174–432) lacking the destruction box was expressed in *E. coli* and co-purified with phosphorylated CDK2 on glutathione sepharose resin (GE Healthcare) (Brown et al., 1999; Welburn and Endicott, 2005). The complex was eluted through GST digestion on column by Prescission protease in 50 mM Tris pH 7.0, 150 mM NaCl, 1 mM DTT buffer. The complex was then buffer-exchanged in phosphorylation buffer (50 mM Hepes, KOH pH 7.8, 12.5 mM MgCl_2_, 1 mM EDTA, 5 mM DTT), concentrated at 46 μM (as determined by Bradford assay), frozen in liquid nitrogen and stored at −80°C until use.

Tau proteins (Tau or Tau-G) were incubated at a final concentration of 0.1 mM with the recombinant CDK2/cyclinA3 complex and 5 mM ATP in phosphorylation buffer at 22°C overnight. After incubation, the kinase was removed by a heating step at 80°C for 15 min and subsequent centrifugation. Tau proteins were then buffer-exchanged in 50 mM ammonium bicarbonate prior to lyophilization. Overall phosphorylation levels were determined by MALDI-TOF mass spectrometry analyses considering a mass increment of +80 Da per phosphate group. Tau or Tau-G proteins phosphorylated by CDK2 were hereafter named Tau-P or Tau-G/P, respectively (Table 1).

### Phosphorylation of Tau by GSK3β

BL21(DE3) *E. coli* strains transformed with a pGEX vector (GE Healthcare) carrying the human *gsk3*β were grown in 1L LB at 37°C for 3 hours until OD at 600 nm reached 0.8–0.9, then the culture was cooled down to 20°C and protein induction was performed at 20°C overnight upon addition of 0.2 mM IPTG. Harvested cells were resuspended in 40 ml extraction buffer (PBS, 10% glycerol, 1% Triton X-100, 10 mM EDTA, 2 mM DTT complemented with protease inhibitor cocktail) and cell lysis was performed by high pressure homogenization. Soluble proteins were isolated by centrifugation at 30,000 × *g* for 30 min at 4°C followed by purification on 1 ml of glutathione sepharose resin (GE Healthcare) per liter of culture. Resin beads were incubated with the soluble extract at 4°C for 3 hours and extensively washed with the extraction buffer, then with phosphorylation buffer supplemented with 50% glycerol. The GST-GSK3β fusion protein on resin beads was stored at −20°C until further use.

Tau proteins (Tau, Tau-G, Tau-P or Tau-G/P) were incubated at final concentrations of 0.1 mM with the recombinant GSK3β kinase (the beads of GST-GSK3β kinase were washed with phosphorylation buffer for glycerol removal) and 5 mM ATP at 30°C overnight in phosphorylation buffer. After incubation, resin beads were removed by centrifugation and heating of the supernatant was performed at 80°C for 15 min followed by centrifugation. Tau proteins were then buffer-exchanged in 50 mM ammonium bicarbonate prior to lyophilization. Overall phosphorylation levels were determined by MALDI-TOF mass spectrometry analyses. Tau, Tau-G, Tau-P or Tau-G/P proteins phosphorylated by GSK3β were hereafter named Tau-P(GSK3), Tau-G/P(GSK3), Tau-PP or Tau-G/PP, respectively (Table 1).

### Peptide Synthesis, Purification, and Characterization

All reagents, amino acids, and solvents were purchased from commercial suppliers and used without further purification if not further mentioned. Solvents- acetonitrile, dimethylchloride, dimethylformamide (DMF)- were purchased from ACROS ORGANICS. Peptides were synthesized on SyroXP-I peptide synthesizer (Multi-SynTech, Witten, Germany) according to standard Fmoc/tBu chemistry using O-(benzotriazol-1-yl)-N,N,NORGANICS. Peptides were synthesiz-phosphate (HBTU)/ hydroxybenzotriazole (HOBt) and preloaded resins (Novabiochem). Fmoc-Ser/Thr(PO(OBzl)OH)-OH (Bachem) was activated with N-[(Dimethylamino)-1H-1,2,3-triazolo-[4,5-b]pyridin-1-ylmethylene]-N-methylmethanaminium hexafluorophosphate N-oxide (HATU)/ diisopropylethylamine (DIPEA) and coupled manually to the resin. DIPEA was added in 3-fold excess with respect to the amino acid and HATU. The reaction time was extended to 6 hours. The coupling of Fmoc-Ser(β-D-GlcNAc(Ac)_3_)-OH was carried out with 2 eq. of the amino acid, HBTU, HOBt and DIPEA in DMF over a time of 6 h (Schwagerus et al., 2016; Reimann et al., 2017). A mixture of 1,8-diazabicyclo[5.4.0]undec-7-ene (DBU) and piperidine (2% each) in DMF was used for Fmoc deprotection. Peptides were cleaved from the resin by treatment with trifluoroacetic acid (TFA)/triisopropylsilane (TIS)/H_2_O (95/2.5/2.5) for 3 hours followed by precipitation with cold diethyl ether.

Purification was carried out by preparative reversed phase HPLC on a Knauer Smartline system (Knauer, Berlin, Germany) equipped with a Luna C8 column (10 μm, 250 × 21.20 mm; Phenomenex, Torrance, CA, United States) running with acetonitrile/0.1% TFA and water/0.1% TFA gradient at 20 mL/min. Purified peptides were characterized by analytical HPLC and high resolution MS. The analytical HPLC was carried out with a VWR-Hitachi Elite LabChrom system (VWR, Darmstadt, Germany) equipped with a Luna C8 column (5 μm, 250 × 4.6 mm; Phenomenex, Torrance, CA, United States). Peptide mass to charge ratios were measured by using an Agilent 6210 ESI-TOF (Agilent Technologies, Santa Clara, CA, United States) (Table 1).

### NMR Spectroscopy

NMR experiments were performed at 293K on a Bruker 600 MHz SB Avance III HD and 900 MHz Avance NEO spectrometer (Bruker, Karlsruhe, Germany) equipped with 5-mm cryogenic triple resonance probe heads. For NMR experiments, ^15^N-labeled proteins and unlabeled peptides were dissolved at 0.2 mM and 2 mM, respectively, in a buffer containing 25 mM NaH_2_PO_4_/Na_2_HPO_4_ pH 6.6, 25 mM NaCl, 2.5 mM EDTA, 1 mM DTT and 5% D_2_O either in a volume of 200 μl (in 3 mm tubes) or in 300 μl (in Shigemi tubes). All ^1^H spectra were calibrated with 1 mM sodium 3-trimethylsilyl-3,3’,2,2’-d4-propionate as a reference. ^1^H spectra were acquired with 64 scans and 32 dummy scans, and a spectral windows of 14 ppm centered on 4.7 ppm sampled with 32k points. For 2D and 3D experiments, a spectral window of 14 ppm centered on 4.7 ppm was used for the proton dimension.

For peptide assignment, standard NOESY and TOCSY experiments were recorded on peptides with 200 and 69 ms mixing times respectively, with 4096 and 512 points and using a DIPSI2 sequence for mixing. TOCSY and NOESY spectra were recorded with 32 scans and 64 scans per increment (and 128 dummy scans), respectively, with spectral windows of 13.98 and 10.0 ppm in each proton dimension centered on 4.69 ppm. ^1^H-^15^N HSQC spectra were recorded at nitrogen-15 natural abundance with 128 scans per increment, with 2048 and 128 points in the proton and nitrogen dimensions, respectively, and with a window of 25 ppm centered on 118 ppm for the nitrogen dimension. ^1^H-^13^C HSQC spectra were recorded with 32 scans per increment, with 1440 and 512 points in the proton and carbon dimensions, respectively, and with a window of 80 ppm centered on 45 ppm for the carbon dimension.

For ^15^N-labeled proteins, ^1^H-^15^N HSQC spectra were recorded with 32 scans per increment and 32 dummy scans with 3072 and 512 points in the proton and nitrogen dimensions, respectively, and with a window of 25 ppm centered on 118 ppm for the nitrogen dimension. Heteronuclear experiments were recorded with a WATERGATE sequence for water suppression and a double INEPT (INsensitive nuclei Enhanced by Polarization Transfer) for sensitivity improvement. All experiments were acquired with a recycle delay of 1s.

Assignment of phosphorylation sites in CDK2/GSK3β phosphorylated TauS262A protein required the acquisition of three-dimensional NMR experiments on ^15^N/^13^C-labeled Tau sample at 293K. The HNCACB and HN(CO)CACB experiments were recorded with non-uniform sampling with 32 scans per increment and 16 dummy scans, with 1540, 90 and 180 points in the proton, nitrogen and carbon dimensions, respectively, and with a window of 25 ppm and 60 ppm centered on 118 ppm and 44 ppm for the nitrogen and carbon dimensions, respectively. The HN(CA)NNH experiment was recorded with non-uniform sampling with 24 scans per increment and 16 dummy scans, with 2048, 120 and 100 points in the proton and both nitrogen dimensions, respectively, and with a window of 25 ppm centered on 118 ppm for the nitrogen dimensions.

*O*-GlcNAc and phosphorylation levels were determined for individual Ser/Thr residues based on intensity of their respective amide correlations of modified and non-modified isoforms from the ^1^H-^15^N HSQC experiment. Resonance assignment, peak intensity and integration are given for phosphorylation and/or *O*-GlcNAcylation sites in TauPHF1 proteins (see Supplementary Table 1). Percentage of phosphorylation for a given residue was calculated as the peak intensities corresponding to phospho isoforms *I(p)* on the sum of total peak intensities including non-modified *I(np/ng)* and *O*-GlcNAcylated *I(g)* isoforms as described in Equation (1). Percentage of *O*-GlcNAcylation is calculated similarly as described in Equation (2). Note that modification of a given residue could affect resonances of vicinal residues leading to resonance splitting, each corresponding to a specific isoform (as exemplified by S400 and S404). Therefore, *I(p)*, *I(g)* or *I(np/ng)* corresponds to the sum of peak intensities of a given residue in its phosphorylated, *O*-GlcNAcylated or non-modified state, respectively, which could occur as several resonances depending on the modification state of its neighbors.

(1) $$\%\mathrm{phosphorylation}=\frac{I\,\left(p\right)}{I\,\left(p\right)+I\,\left(n\,p/n\,g\right)+I\,\left(g\right)}$$

(2) $$\%O-\text{GlcNAcylation}=\frac{I\,\left(g\right)}{I\,\left(p\right)+I\,\left(n\,p/n\,g\right)+I\,\left(g\right)}$$

The levels of phosphorylation or *O*-GlcNAcylation can either be determined using peak integration to take into account changes of relaxation of amide ^1^H and ^15^N upon modification. However, overlapping peak could lead to misinterpretation of modification levels. A comparison of values obtained from peak intensities and peak integrals gives similar results (see Supplementary Table 2).

### MALDI-TOF Mass Spectrometry

Tau proteins were analyzed by MALDI-TOF MS (Axima Assurance, Shimadzu) in a linear positive ion mode with sinapinic acid matrix after ZipTip^®^-C4 desalting (Millipore). For the calculation of overall phosphorylation and *O*-GlcNAcylation levels, m/z increments of +80 Da and +203 Da were used, respectively.

### Aggregation of Tau Proteins

Aggregation reactions were performed into 96-well black plate (Greiner) in a plate reader (PHERAStar, BMG Labtech) at 37°C without agitation. A 5 mM aqueous stock solution of ThT (Sigma) was filtered through a 0.22μm-filter. Tau aggregation was induced by heparin (as sodium salt from porcine intestinal mucosa, polydisperse heparin with molecular weight range 6–30 kDa with most chains in the 17–19 kDa range; Sigma) and kinetics were measured for 5–7 days with 10 μM Tau and 2.5 μM heparin (ratio 4:1) in a buffer containing 25 mM NaH_2_PO_4_/Na_2_HPO_4_ pH 6.6, 25 mM NaCl, 2.5 mM EDTA, 0.33 mM DTT, and 50 μM ThT. Kinetics of fibril formation were monitored by ThT fluorescence emission at 490 nm (with excitation at 440 nm). Aggregation reactions of each Tau PTM isoform were performed in duplicate or triplicate and kinetics curves represented as mean ± standard deviation were fitted with a model of single-phase association with the GraphPad Prism software. Different batches of kinase and phosphorylated Tau were prepared and involved in aggregation reactions with kinetics curves similar as those depicted in Figure 5 except for the plateaus that differ between batches. Measurements of decrease in soluble Tau monomers upon aggregation was performed at end-point on SDS-PAGE (4–20% polyacrylamide) by comparing the protein amounts before and after aggregation (equivalent of 5μg of monomeric Tau were loaded in each lane) using the ImageJ software (Abramoff et al., 2004). Observation of fibril morphology was performed at the end of aggregation reactions by transmission electron microscopy (Zeiss EM 900 microscope at 80kV equipped with a Gatan Orius 1000 camera). Two grids per sample were prepared with 10μl of aggregation reaction that were applied on 400-mesh hexagonal formvar-coated grids for 90 sec. The sample-loaded grids were washed three times with ultrapure water and drained. The grids were then negatively stained with 2% uranyl-acetate solution for 90 sec and washed twice with ultrapure water. All TEM images were selected as representative for each experimental condition (i.e., Tau isoform).

For aggregation reactions induced by seeding, seeds were prepared by incubating TauS262A (in its non-modified form) at 10 μM and heparin at 2.5 μM (ratio 4:1) for 7 days at 37°C. After sonication, the aggregation reaction was diluted at 1:10^e^ in fresh monomeric Tau at 25 μM in 100 mM MES pH 6.9, 2mM EGTA, 1mM MgCl_2_, 20 mM NaF, 1 mM DTT, 100 μM ThT. Control of seeds without fresh monomeric Tau was performed in the same conditions. As a control of the absence of free heparin, TauS262A protein at 25 μM was incubated with 0.25 μM heparin (ratio 100:1) in the same conditions than the seeding reactions. Seeding reactions were incubated for a total of 5 to 7 days at 37°C. Negative-staining TEM imaging and SDS-PAGE measurement of decrease in soluble monomer concentration upon assembly were performed at the aggregation end-point as described for heparin-induced aggregation assays.

## Results

### Phosphorylation and *O*-GlcNAcylation Crosstalk in Tau Protein

#### GSK3β Phosphorylation of Tau With and Without Priming by CDK2/Cyclin A

We aim to investigate how *O*-GlcNAcylation modulates phosphorylation of Tau by GSK3β with or without priming by the proline-directed kinase CDK2/cyclin A3 (referred hereafter as CDK2). As major *O*-GlcNAc sites were all found in the C-terminus of Tau (see Supplementary Figures 1A,B) (Bourré et al., 2018), we focused the study of PTM crosstalk between phosphorylation and *O*-GlcNAcylation in a mutated form of Tau – TauPHF1– in which most of Ser/Thr proline-directed phosphorylation sites except those of the PHF-1 epitope were mutated into Ala residues (see Supplementary Figure 2). Mutations in TauPHF1 allowed to simplify the phosphorylation pattern for further functional studies of the PHF-1 epitope’s role in Tau aggregation. The PTM patterns of TauPHF1 were compared to those of the TauS262A mutant protein in which S262 mutation into alanine prevents inhibition of Tau aggregation by S262 phosphorylation (although phosphorylation of S262 is expected here neither with CDK2 nor GSK3β activity). TauS262A protein exhibits a more complex phosphorylation pattern due to the presence of 17 Ser/Thr-Pro motifs targeted by proline-directed kinases such as CDK2 (Amniai et al., 2009; Landrieu et al., 2010).

**FIGURE 2:**
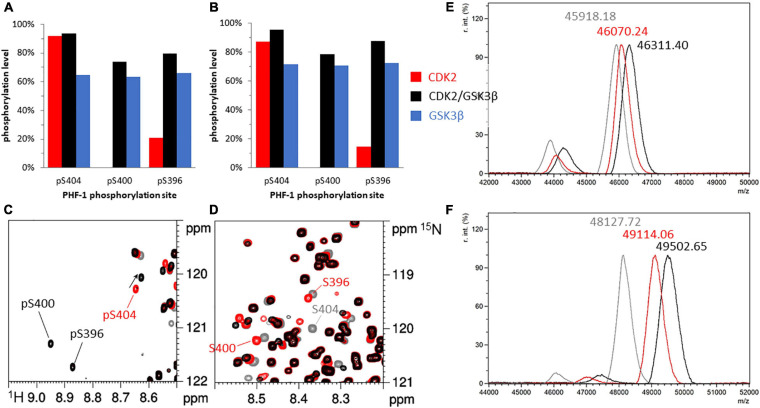
Phosphorylation patterns of ^15^N-TauPHF1 **(A,C,D,E)** and ^15^N^13^C-TauS262A **(B,F)** by GSK3β with and without priming by CDK2. **(A,B)** Phosphorylation levels of the PHF-1 epitope phosphorylated by CDK2 (red bars), CDK2/GSK3β (black bars) and GSK3β without CDK2 priming (blue bars). **(C,D)** ^1^H-^15^N HSQC spectra of TauPHF1 in its non-phosphorylated form (gray), phosphorylated by CDK2 (red) and by CDK2/GSK3β (black) showing the resonances of PHF-1 residues corresponding to the phosphorylated state **(C)** and the non-phosphorylated state **(D)**. **(E,F)** Mass spectrometry analyses of ^15^N-TauPHF1 **(E)** and ^15^N^13^C-TauS262A **(F)** with the same colors as NMR spectra depicted in **(C,D)**.

To establish baseline conditions, we first compare GSK3β phosphorylation with or without priming by CDK2 in non-*O*-GlcNAcylated Tau mutants, using mass spectrometry and NMR spectroscopy (Smet et al., 2004a; Lippens et al., 2006, 2016; Landrieu et al., 2010; Danis et al., 2016, 2019). Without priming by CDK2, only few phosphorylations were detected in the ^1^H-^15^N HSQC spectrum of TauS262A (see Supplementary Figures 3B,D). Resonances corresponding to the amide group of phospho-residues exhibiting downfield ^1^H chemical shifts as compared to their non-phosphorylated counterparts were the same in both TauS262A and TauPHF1 mutants indicating that GSK3β-mediated phosphorylation is restrained to residues of the PHF-1 epitope when Tau was not primed by CDK2 (see Supplementary Figures 3A,B). Mass spectrometry analyses of TauPHF1-P(GSK3) indicate a m/z increment of +218.0 ± 52 Da as compared to the non-phosphorylated protein corresponding to an average of 2.7 ± 0.6 phosphate per Tau molecule. The same overall phosphorylation level was measured for TauS262A-P(GSK3) (see Supplementary Figures 3C,D). Assignment of phospho-residue resonances allowed both the identification of phosphorylation sites and the quantification of phosphorylation levels in a site-specific manner (Landrieu et al., 2010; Theillet et al., 2012; Danis et al., 2016, 2019). Single resonances were detected for each phospho-residue (S396, S400, S404) (Figure 2C, see Supplementary Figures 3A,B) together with similar phosphorylation levels of 72% ± 2% for TauS262A and 65% ± 2% for TauPHF1 (Figures 2A,B). These data indicate a homogenous pattern of phosphorylation corresponding to the triple phosphorylated state of the PHF-1 epitope, hereafter named PHF1-3P.

In contrast, CDK2 priming prior to GSK3β phosphorylation of TauS262A and TauPHF1 led to distinct phosphorylation patterns (Figures 2E,F). CDK2-mediated phosphorylation of TauS262A (annotated by TauS262A-P) occurred at several sites in the proline-rich region at T153, T175, T181, S199, S202, T205, T212, T231, S235 residues (see Supplementary Figures 4A,B), upstream the MTBD, and at S396 and S404 within the C-terminal domain while only S396 and S404 were found in TauPHF1, as expected (Figures 2A–C and Supplementary Figures 4A–C). Mass spectrometry analyses of TauS262A-P indicate a m/z increment of +986.34 ± 64Da as compared to the non-phosphorylated protein corresponding to an average of 12.3 ± 0.8 phosphate per Tau molecule (Figure 2F). Subsequent phosphorylation by GSK3β (annotated by TauS262A-PP) is modulated by CDK2 priming. A m/z increment of +1374.93 ± 61 Da as compared to the non-phosphorylated protein corresponding to an average of 17.2 ± 0.8 phosphate per Tau molecule was found indicating an addition by GSK3β of about 5 ± 0.8 phosphate per Tau (Figure 2F). In TauS262A-PP, three new phosphorylation sites were identified at S198 and S208 in the PRR resulting from priming at S202 and T212, respectively, and S400 in the C-terminus resulting from priming at S404 while S396 phosphorylation level is strongly increased. Phosphorylation by GSK3β after CDK2 priming leads to a triple phosphorylation state of both AT8 (pS202/pT205/pS208, AT8-3P) and PHF-1 epitopes (Figures 2B,F and Supplementary Figures 4A,B). At the PHF-1 epitope, CDK2 phosphorylation reached 85% and 15% on S404 and S396, respectively. After GSK3β phosphorylation, S404, S400 and S396 were phosphorylated at a level of 95%, 80% and 88%, respectively, leading to an overall level of 80% for the PHF1-3P epitope (75% in TauPHF1) considering a sequential phosphorylation mechanism (Figures 2A,B, see Supplementary Figure 4E). Together, these data indicate that PHF-1 epitope is unique as GSK3β substrate within Tau sequence as the only epitope that does not require priming by another kinase. In contrast, AT8-3P required priming at T212 by CDK2, or another proline-directed kinase, prior to GSK3β phosphorylation.

#### GSK3β Phosphorylation of *O*-GlcNAcylated Tau With and Without Priming by CDK2/Cyclin A

In a similar manner described for TauS262A and TauPH1, GSK3β phosphorylation of *O*-GlcNAcylated forms was investigated with and without CDK2 priming. In accordance with what we have previously described (Bourré et al., 2018), when incubated with full-length Tau, recombinant OGT (in its nucleocytoplasmic isoform referred as ncOGT) provides three major *O*-GlcNAc sites that are all found in the C-terminus at residues S400, S412 and S413. These *O*-GlcNAcylated Tau proteins are hereafter named Tau-G (TauS262A-G and TauPHF1-G). As the quantitative pattern of *O*-GlcNAcylation is dependent on the conditions used for the *O*-GlcNAc transferase reaction, we achieve a high level of *O*-GlcNAc modification by reverse phase high-performance liquid chromatography enrichment of *O*-GlcNAcylated species in order to unambiguously detect perturbations of phosphorylation patterns induced by *O*-GlcNAcylation. This procedure leads to an overall *O*-GlcNAc level of 1.8 ± 0.3 GlcNAc per Tau molecule and site-specific *O*-GlcNAcylation of 61% ± 2%, 27% ± 1% and 47% ± 2% on S400, S412 and S413, respectively, determined by high resolution NMR spectroscopy on both TauS262A-G and TauPHF1-G (see Supplementary Figures 1A,B,E,F).

**FIGURE 3:**
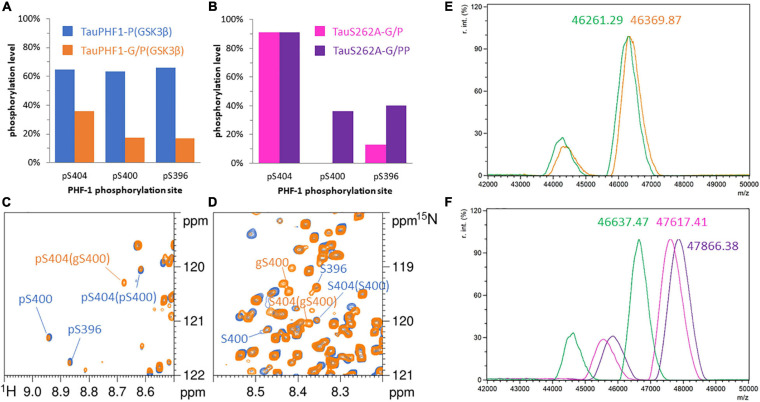
Phosphorylation patterns of ^15^N-TauPHF1-G **(A,C,D,E)** by GSK3β without priming by CDK2 and ^15^N-TauS262A-G **(B,F)** by GSK3β with CDK2 priming. **(A,B)** Phosphorylation levels of the PHF-1 epitope. **(A)** TauPHF1 (blue bars) and TauPHF1-G (orange bars) phosphorylated by GSK3β. **(B)** TauS262A-G phosphorylated by CDK2 (pink bars) and GSK3β after CDK2 priming (purple bars). **(C,D)** ^1^H-^15^N HSQC spectra of TauPHF1 (blue) and TauPHF1-G (orange) phosphorylated by GSK3β without priming by CDK2 showing the resonances of PHF-1 residues corresponding to the phosphorylated state **(C)** and the non-phosphorylated state **(D)**. Resonances related to the *O*-GlcNAcylated forms of S400 is indicated by “gS400”. **(E,F)** Mass spectrometry analyses of ^15^N-TauPHF1-G (E) before (green) and after GSK3β phosphorylation (orange) and ^15^N-TauS262A-G (F) before (green) and after CDK2 (pink) or CDK2/GSK3β (purple) phosphorylation.

GSK3β-mediated phosphorylation of TauPHF1-G drop down to an average of 1.3 ± 0.6 phosphate per Tau molecule (Figure 3E) with a phosphorylation level of 38% on S404 and 14% on both S400 and S396 (Figure 3A). Three resonances were detected for the amide function of pS404 isoforms in TauPHF1-G/P(GSK3β) representing 21%, 14% and 3% of the total population and were assigned to pS404/gS400, pS404/pS400 and pS404/S400 isoforms (Figure 3C), respectively, based on comparison with spectra of TauPHF1-P and TauPHF1-PP in which resonance of pS404/S400 and pS404/pS400 were found, respectively (Figure 2C). In contrast, *O*-GlcNAcylation did not affect S404 and S396 phosphorylation by CDK2 (90% and 15%, respectively). However, subsequent GSK3β phosphorylation is significantly reduced as compared to non-*O*-GlcNAcylated Tau to ca. 36% and 40% on both S400 and S396 sites, respectively (Figure 3B).

Hence, *O*-GlcNAcylation reduces GSK3β phosphorylation on PHF-1 epitope in which S400 *O*-GlcNAcylation is likely involved. Overall phosphorylation of PHF1-3P is reduced from 80 to 36% approximately upon sequential activity of CDK2 and GSK3β, and from 65 to 14% in the absence of CDK2 priming. In TauPHF1-G/P(GSK3β), the overall S404 phosphorylation level of 38% is distributed between three isoforms: 14% for PHF1-3P, 21% for gS400/pS404 and 3% for S400/pS404, the last two being not subjected to further phosphorylation at S400 and S396 (Figure 3C). These data indicate that phosphorylation and *O*-GlcNAcylation of respective S404 and S400 sites are not exclusive but rather competitive. The non-phosphorylated S404 isoform contains a higher proportion of gS400 (75%) than the pS404 isoform (55%) indicating that *O*-GlcNAcylation is preferentially associated to the non-phosphorylated isoform.

Furthermore, levels of 26% and 38% for pS400/pS404 and gS400/pS404 isoforms, respectively, were expected considering a S404 phosphorylation level of 65% (as PHF1-3P) and S400 *O*-GlcNAcylation level of 60%. Lower values were measured indicating that *O*-GlcNAcylation not only reduces S404 phosphorylation by GSK3β (by two-fold) and directly prevents formation of PHF1-3P epitope, but also reduces S404 phosphorylation in the S400 non-*O*-GlcNAc fraction indicating a potential role of *O*-GlcNAcylation at S412 and S413 sites in modulation of PHF-1 epitope phosphorylation.

In contrast, remote phospho-epitopes localized in the proline-rich domain of TauS262A were not regulated by *O*-GlcNAc modifications within the C-terminal domain. Therefore, *O*-GlcNAcylation neither modulates CDK2 priming nor has a long-range influence on phosphorylation sites localized in the proline-rich domain.

### PTMs of PHF-1 Epitope Modulate Local Conformation of Tau Peptides

**FIGURE 4:**
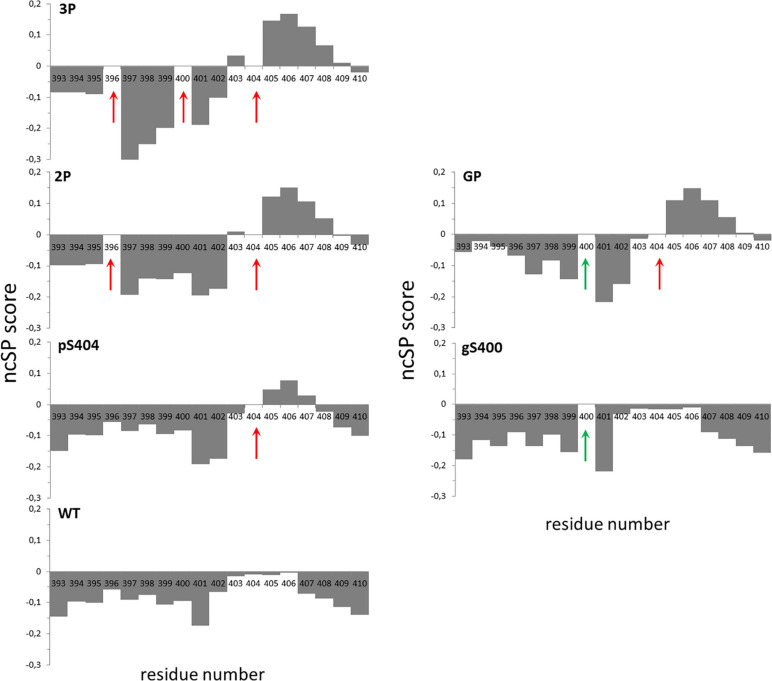
Neighbor-corrected structural propensity (ncSP) plots of Tau[392-411] peptide series indicating the impact of multiple PTMs on peptide conformation (Tamiola and Mulder, 2012). Phospho and *O*-GlcNAc PTM sites are indicated by red and green arrows, respectively. Deviations of combined ^1^Hn, ^15^N, ^1^Hα, ^13^Cα and ^13^Cβ chemical shifts of the WT (without PTM), pS404, gS400, 2P, 3P and GP peptides compared with neighbor-corrected IDP chemical shift library (Tamiola and Mulder, 2012) illustrates conformational changes of peptide C-terminus upon pS404 which is enhanced by combination with either multiple phosphorylations or *O*-GlcNAcylation.

The effect of phosphorylation, *O*-GlcNAcylation and combination of both PTMs on Tau conformation was investigated by high-resolution NMR spectroscopy (see Supplementary Figures 5A,B) in small peptides centered on the PHF-1 epitope from amino acid 392 to 411 (according to numbering of the longest Tau2N4R isoform of 441 residues, hereafter named Tau[392-411], Table 2). Here, solid-phase peptide synthesis allows the introduction of PTMs in a site-specific manner providing homogenous modification patterns. All peptides exhibit random coil conformations as shown by ^13^C secondary chemical shifts of Cα and Cβ (see Supplementary Figure 6A). Peptides without PTM or with S400-*O*-GlcNAc have both a weak propensity of secondary structure along the entire sequence as shown by neighbor-corrected Structural Propensity (ncSP) calculation (Figure 4) (Tamiola and Mulder, 2012). In contrast, peptides with phosphorylation on S404 have a slight propensity to adopt helical conformation in the C-terminal region (from S404 to L408) as highlighted by both secondary structure propensity (SSP) based on ^1^Hα, ^13^Cα and ^13^Cβ (Supplementary Figure 6B) (Marsh et al., 2006) and ncSP scores based on ^1^H_N_, ^15^N, ^1^Hα, ^13^Cα and ^13^Cβ (Figure 4). This structural propensity is more pronounced in the presence of other PTMs, either phosphorylation of S396 (Tau[392-411]-2P) or double phosphorylation of S396/S400 (Tau[392-411]-3P) or glycosylation of S400 (Tau[392-411]-GP) while the N-terminal region (from I392 to D402) have an even more pronounced extended conformation upon multiple PTMs (Figure 4). Conformational changes induced by pS404 and enhanced by multiple PTMs are also evidenced by the extent of perturbations of amide and Hα-Cα chemical shifts from R406 to N410 (see Supplementary Figures 5B, 7A,B). Together, these data suggest that phosphorylation of S396, and phosphorylation or *O*-GlcNAcylation of S400 have a remote effect on the conformation of the region immediately downstream pS404 inducing a propensity to helical structure probably stabilized by H-bond and/or salt bridges between pS404 and basic residues within the C-terminus.

**TABLE 2:** Primary structure of the Tau-PHF1 peptide series and posttranslational modification sites.

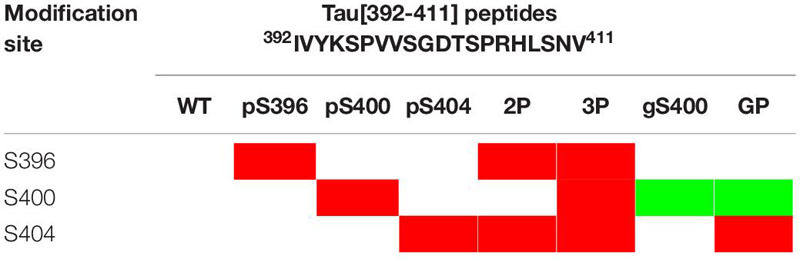

*Phosphorylation are indicated in red and *O*-GlcNAcylation in green.*

### Effect of Phosphorylation and *O*-GlcNAcylation of PHF-1 Epitope on the Formation of Tau Fibrillar Aggregates

**FIGURE 5:**
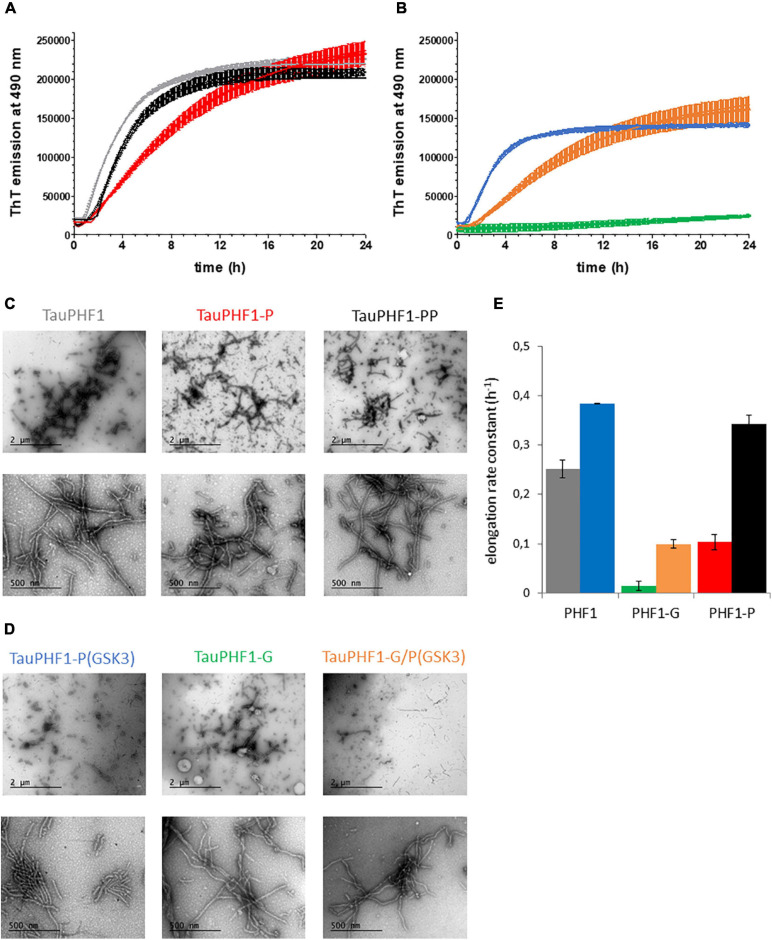
Aggregation of TauPHF1 series induced by heparin. **(A,B)** Kinetics of TauPHF1 aggregation probed by ThT fluorescence emission plotted as mean ± standard deviation. **(C,D)** TEM images showing the fibril morphology of TauPHF1 isoforms with 20,000× (upper panels) and 85,000× (lower panels) magnification. The scale bars of the upper panels correspond to 2 μm and for the lower panels to 500 nm. Isoforms that are non-phosphorylated by GSK3β (Tau, Tau-G, and Tau-P) and phosphorylated by GSK3β (Tau-P(GSK3), Tau-G/P(GSK3), and Tau-PP) are depicted in **(C)** and **(D)**, respectively. **(E)** Elongation rate constants calculated from curves fitted with a one-phase association model. The color coding is the same for all panels as indicated in **(C)** and **(D)** for the different TauPHF1 isoforms.

The effect of PTMs within the PHF-1 epitope was then investigated at the functional level in Tau self-assembly into fibrillar aggregates. Without external inducer, neither TauS262A-P(GSK3), TauPHF1-P nor TauPHF1-PP were able to form fibrils as monitored by Thioflavin T (ThT) fluorescence during several days at 37°C and negative-staining transmission electron microscopy (TEM). Hence, we first stimulated aggregation of the diverse phosphorylated/*O*-GlcNAcylated Tau isoforms by using heparin and tracking aggregation kinetics by ThT fluorescence combined with TEM imaging at the end-point of aggregation to probe fibril morphology and extent. We have previously shown that heparin as a polyanionic cofactor mainly interacts with lysine residues in Tau protein with higher avidity for MTBR and flanking regions (Sibille et al., 2006; Kamah et al., 2014). Hence, multiple mutations of Ser/Thr into Ala residues did not significantly affect heparin binding to TauPHF1 mutant which forms fibrillar aggregates with kinetics similar to TauS262A (Figure 5A,D, see Supplementary Figures 8A, 9A). We have observed the formation of filaments for the various PTM isoforms of TauPHF1 with morphology similar to PHFs as detected by TEM (Figure 5D). However, it has been described that heparin-induced fibrils of Tau are polymorphic and morphologically distinct from those extracted from AD brain (Fichou et al., 2018d; Zhang et al., 2019). As the molecular structure of fibrils from Tau PTM isoforms may be different from *bona fide* PHFs, we hereafter referred them to PHF-like fibrils.

Formation of filaments of TauPHF1 proteins was modulated by the different PTMs. Notably, *O*-GlcNAcylation reduces the elongation rate of TauPHF1 as previously shown (Yuzwa et al., 2012). However, ThT signal of TauPHF1-G was increased significantly at end-point of aggregation reactions although about 3 times lower than TauPHF1 (Figures 5A,B, see Supplementary Figures 8A–C). TEM examination and measurement of variations in the amount of soluble TauPHF1-G before and at the end of aggregation indicate a high content of fibrils together with a significant decrease of soluble protein suggesting that *O*-GlcNAcylation does not prevent fibrillar assembly of Tau (Figures 5B,D, see Supplementary Figures 8D,E). While both CDK2 and sequential CDK2/GSK3β phosphorylation strongly decrease TauS262A aggregation, aggregation of TauPHF1 was modulated by the sole phosphorylation of PHF-1 epitope in different way depending on kinase involved: CDK2 phosphorylation decreases the rate of fibril formation while GSK3β phosphorylation stimulates aggregation of all proteins - Tau-P(GSK3), Tau-PP and Tau-G/P(GSK3) - as shown by comparing the elongation rate constants derived from aggregation kinetics with values of Tau proteins before GSK3β phosphorylation - Tau, Tau-P and Tau-G, respectively (Figure 5C). GSK3β phosphorylation after CDK2 priming or without priming leads to similar patterns of phosphorylation in TauPHF1 mutant primarily defined by a high level of PHF1-3P (Figure 2A) and same aggregation kinetics of PHF-like fibril formation (Figures 5A–C). In contrast, even though GSK3β phosphorylation stimulates aggregation of Tau-G, it does not restore the aggregation rate at the level of non-modified Tau but rather parallel the aggregation kinetics of Tau-P. Together, these data indicate that the PHF1-3P isoform plays an active role in Tau aggregation and *O*-GlcNAcylation although decreasing the elongation rate and modulating the extent of PHF1-3P isoform interferes only weakly with the fibrillar assembly of phosphorylated Tau.

**FIGURE 6:**
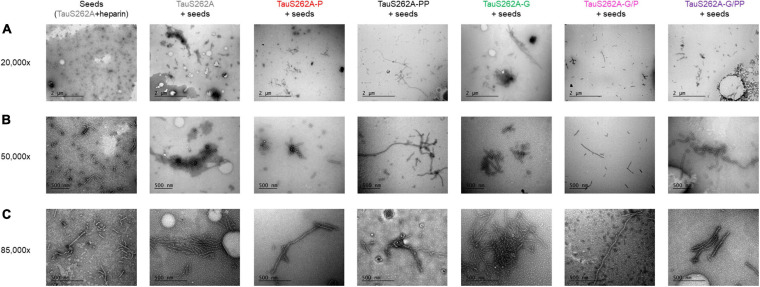
Transmission electron micrographs with negative staining of fibrillar aggregation of TauS262A protein series induced by Tau seeds prepared with TauS262A and heparin (ratio 4:1) for 7 days at 37°C, diluted 10-fold in 25 μM monomeric Tau (gray), Tau-P (red), Tau-PP (black), Tau-G (green), Tau-G/P (pink) and Tau-G/PP (purple) showing three different magnification: 20,000× **(A)**, 50,000× **(B)** and 85,000× **(C)**. The scale bar of 2 μm applies to panels **(A)**, 500 nm to panels **(B,C)**.

TauS262A mutant phosphorylated by CDK2 or sequentially by CDK2 and GSK3β (TauS262A-P or TauS262A-PP), both providing high levels of phosphorylation, only marginally forms fibrillar aggregates using heparin as external inducer as compared to the non-phosphorylated isoform (see Supplementary Figures 9A,B). Phosphorylation, either high level or site-specific, probably competes with heparin binding making it impossible to use and meaningless. Along this line, other PTM of Tau could also modulate fibril formation by interfering with inducer binding complicating data interpretation. To circumvent issues related to potential reduction of heparin binding upon phosphorylation and/or *O*-GlcNAcylation, we have then investigated aggregation induced by seeds formed with TauS262A in the presence of heparin. ThT fluorescence was only weakly increased in aggregation reactions of soluble, monomeric Tau proteins at 25 μM in the presence of a 10-fold dilution of seeds rendering difficult the interpretation and comparison of kinetics parameters (see Supplementary Figures 10A,B). However, observation of PHF-like fibrils by TEM clearly indicate an increase of the fibril content and length in the presence of monomeric Tau proteins bearing diverse PTMs as compared to seeds alone. A high content of isolated and clusters of long PHF-like fibrils were observed for both TauS262A and TauS262A-G proteins while only individual short-length fibrils were detected for seeds alone (Figure 6). In contrast, phosphorylated forms of TauS262A and TauS262A-G prevents the formation of clusters of fibrils and promote rather individual PHF-like filaments longer than those observed in seeds alone. In TauPHF1 isoforms, phosphorylation by GSK3β of either Tau, Tau-P or Tau-G promotes the formation of large fibrillar clusters constituted of long PHF-like filaments (see Supplementary Figure 11A).

## Discussion

We have investigated phosphorylation of Tau by GSK3β with and without priming by CDK2, with and without prior *O*-GlcNAcylation by OGT to probe modulation of GSK3β-mediated phosphorylation pattern and its role on Tau self-assembly into PHF-like filaments. The phosphorylation pattern provided by GSK3β in TauS262A is highly regulated by CDK2 priming. While only the triple phosphorylated state of PHF-1 epitope or PHF1-3P (pS396/pS400/pS404) was observed without priming as previously shown (Leroy et al., 2010), prior CDK2 priming provides additional phosphorylation in the PRD. CDK2 phosphorylates the AT180 phospho-epitope (pT231/pS235) and AT8 epitope in its double phosphorylated state or AT8-2P (pS202/pT205). Other phosphorylations were also detected in the PRD (pT153, pT175, pT181, pT212) with pT212 and pS202 that serves as priming sites for phosphorylation by GSK3β of S208 and pS198, respectively, leading to a triple phosphorylated state of AT8 or AT8-3P (pS202/pT205/pS208) (Despres et al., 2017). Together, most of phosphorylation sites were found within the C-terminal half of TauS262A protein with an overall high phosphorylation level of 17 ± 1 phosphate per Tau when sequentially combining CDK2 and GSK3 phosphorylation, in agreement with physiological Tau expressed in Sf9 eukaryotic cells (Drepper et al., 2020).

**FIGURE 7:**
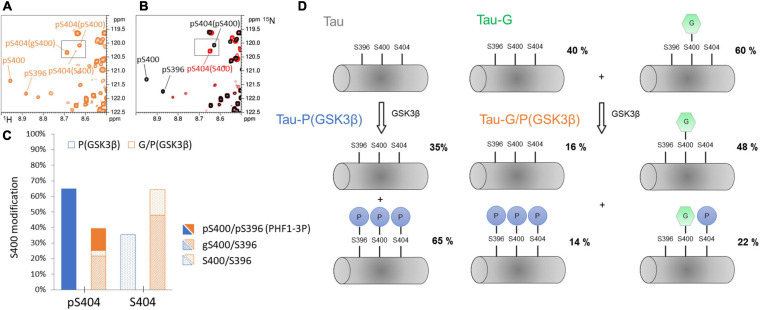
Phosphorylation pattern of GSK3β at the PHF-1 epitope without CDK2 priming and its regulation by *O*-GlcNAc. **(A,B)** overlaid ^1^H-^15^N-HSQC NMR spectra of TauPHF1-G/P(GSK3β) (orange), TauPHF1-P (red), and TauPHF1-PP (black) showing the resonance multiplicity of pS404 depending on the modification state of S400. **(C)** Graphical representation of levels of S400 modifications (S400, pS400, or gS400) depending on the phosphorylation state of S404 in TauPHF1-G/P(GSK3β) (orange bars) as compared to TauPHF1-P(GSK3β) (blue bars). Phosphorylation levels of S400 are depicted by filled areas, *O*-GlcNAcylation levels by shaded areas and no PTM by dotted areas. **(D)** Schematic representation of phospho-/*O*-GlcNAc-isoforms of the PHF-1 epitope with their respective distribution in TauPHF1-G, TauPHF1-P(GSK3β) and TauPHF1-G/P(GSK3β).

On the other hand, *O*-GlcNAcylation by OGT is solely localized within the C-terminus of Tau at S400, S412 and S413 residues. With or without CDK2 priming, OGT glycosylation only regulates phosphorylation of PHF-1 epitope primarily through S400 *O*-GlcNAcylation embedded in that epitope (at a level of ca. 60%), acting by both decreasing phosphorylation on S404 and disrupting sequential phosphorylation of S400/S396 by GSK3β as previously described in peptides (Smet-Nocca et al., 2011) while priming by CDK2 on S404 is not affected. Formation of PHF1-3P by GSK3β without priming by CDK2 was decreased upon *O*-GlcNAcylation from about 65% to 14% (Figures 3A, 7C). Phosphorylation and *O*-GlcNAcylation of respective S404 and S400 sites are not exclusive as they co-exist in an isoform representing 21% of all isoforms. However, the gS400 glycosylation, differentially distributed into pS404 and S404 isoforms, is prevalent in the non-phosphorylated S404 isoform (see Supplementary Figure 12). This is in agreement with what we have previously shown with phosphorylation of Tau-G by the kinase activity of a rat brain extract (Bourré et al., 2018). Furthermore, *O*-GlcNAcylation not only reduces the formation of PHF1-3P epitope but also decreases S404 phosphorylation in the S400 non-*O*-GlcNAc fraction pointing to regulatory role of other *O*-GlcNAc sites at S412 and S413 in modulation of PHF-1 phosphorylation.

From a conformational point of view, our NMR data indicate that amide resonances of pS404 or S404 are sensors of the PTM state of S400, and conversely (Figures 2C,D, 3C,D, and Supplementary Figures 1C,D) underpinning a spatial proximity of both sites and potential conformational crosstalk. The study of local conformational changes induced by PTM of the PHF-1 epitope was performed in small peptides (Table 2 and Supplementary Figures 5A,B) ranging from residue 392 to 411 using NMR chemical shifts. Chemical shift index (Wishart et al., 1992, 1995) and secondary chemical shift (Wishart et al., 1991; Wishart, 2011) analyses based on ^13^Cα and ^13^Cβ indicate a random coil conformation for Tau[392-411] peptides independently of their PTM status (see Supplementary Figure 6A). Chemical shift analyses of Tau[392-411] peptides with SSP and ncSP indicate an extended conformation for the N-terminal region (from residue 392 to 402) while the peptide C-terminus displays conformational changes according to PTM involved. An extended conformation of a di-phosphorylated peptide, pS396/pS404, was revealed by an X-ray structure of the PHF-1 epitope in complex with a monoclonal Tau antibody emphasizing side chain interactions between Y394 and pS396 stabilized by specific interactions with an antigen binding site (Chukwu et al., 2018). In ^1^H-^15^N HSQC spectra of pS396-containing Tau isoforms and peptides, a large shift was observed for K395 resonance upon S396 phosphorylation (see Supplementary Figures 4A,C, 5A, 7B) while perturbations of chemical shifts of surrounding residues such as Y394 are weaker. This suggests that adjacent K395 and pS396 residues engage in a salt bridge as also shown in MD simulations of model peptides of similar sequence surrounding a phospho-site (Rani and Mallajosyula, 2017). It has been proposed that an intramolecular salt bridge between lysine or arginine side chain and phosphate locks the peptide in a Poly-Proline II (PPII) conformation. In RT^231^PP sequence of Tau, a salt bridge interaction between R230 and pT231 was suggested to compete with an intermolecular salt bridge with tubulin (Schwalbe et al., 2015). Similarly, phosphorylation of S396 could lock both K395 and S396 in a salt bridge preventing interactions of these residues with other partners.

On the other hand, chemical shift analyses with SSP and ncSP highlight a helical propensity in the C-terminus of peptides containing phosphorylation at S404 (Figure 4). In contrast, none of individual phosphorylation on S396 or S400 or *O*-GlcNAcylation of S400 induce similar conformational changes (see Supplementary Figure 6B). The S404 phosphorylation-induced conformational change is in agreement with what was previously observed for phosphorylation of AT180 epitope where phosphorylation of T231/S235 induces a N-cap stabilizing a α-helix in the C-terminal region of phosphorylation sites (from S237 to A246) (Bielska and Zondlo, 2006; Sibille et al., 2011). Interestingly, the helical conformation tendency induced by pS404 is exacerbated when other PTMs are present on either S396 and/or S400. Surprisingly, *O*-GlcNAcylation (Tau[392-411]-GP) induces the same effect on the C-terminus conformation as single phosphorylation of S396 (Tau[392-411]-2P) or double phosphorylation of S396/S400 (Tau[392-411]-3P) (Figure 4). It was shown in Tau peptides from PRD that phosphorylation induces the formation of a PPII helix while *O*-GlcNAcylation have an opposite role by destabilizing it (Brister et al., 2014). In a study performed in model α-helical peptides, both phosphorylation and *O*-GlcNAcylation localized at the N-terminus of an α-helix were shown to stabilize it with a greater effect for phosphorylation over *O*-GlcNAcylation, and both destabilize it when localized within the helix sequence with a greater effect for *O*-GlcNAcylation (Elbaum and Zondlo, 2014). Here, in the PHF-1 epitope, phosphorylation of S396 and S400 as well as *O*-GlcNAcylation of S400 are located in an extended N-terminal region with respect to those of helical propensity and remotely stabilize a conformational change induced by phosphorylation of S404. Such conformational changes may have critical implications in interactions of Tau with microtubules or other binding partners, in regulating Tau function of tubulin polymerization or self-assembly into fibrils.

The role of PHF-1 phosphorylation and *O*-GlcNAcylation in fibrillar aggregation was then investigated *in vitro* in the presence of heparin or seeds of Tau2N4R to induce aggregation. High level or site-specific phosphorylation are likely to compete with Tau binding to heparin due to charge repulsion, as illustrated by all phosphorylated isoforms of TauS262A and TauS262A-G (see Supplementary Figures 9A–C). Phosphorylation sites encompassed in the PRD are prone to interfere with heparin binding due to a high proportion of positively charged residues, especially lysine residues, in this region involved in heparin interactions while the C-terminal domain contains a lower concentration of binding sites (Sibille et al., 2006; Kamah et al., 2014; Despres et al., 2019). Hence, phosphorylation of PHF-1 epitope is less likely to interfere with heparin binding than multiple phosphorylation within the PRD. Reducing phosphorylation sites in TauPHF1 mutant leads to appreciable aggregation rates of phosphorylated isoforms as measured by ThT fluorescence and sizable amounts of PHF-like fibrils observed by TEM. In this aggregation set-up in which heparin stimulates the formation of filaments, phosphorylation by CDK2 (Tau-P) as well as *O*-GlcNAcylation by OGT (Tau-G) both reduce fibrillar aggregation rates with a greater effect for *O*-GlcNAcylation, which also affects the initial phase of fibrillization related to nucleation processes (Figure 5, see Supplementary Figure 8). Nevertheless, significant increase of ThT fluorescence and decrease of soluble protein content at aggregation end-point as well as large amounts of PHF-like filaments shown by TEM (Figure 5D, see Supplementary Figure 8) indicate that *O*-GlcNAcylation does not prevent fibrillar assembly of Tau but only reduces the elongation rate.

In contrast, phosphorylation by GSK3β accelerates aggregation rates of Tau, Tau-P or Tau-G isoforms by 1. 5-, 3.3- and 7.1-fold, respectively (Figure 5C). Each of the respective phospho-isoforms resulting from GSK3β phosphorylation contains PHF1-3P epitope at a relative level of 65%, 75% and 14% (Figures 2A, 3A, 7C). Even though Tau-G/P(GSK3β) still contains a significant amount of non-phosphorylated gS400 isoform (48%) that was shown to strongly inhibit the aggregation rate, GSK3β phosphorylation significantly stimulates fibrillar assembly even with a low amount of PHF1-3P. Together these data indicate that the PHF1-3P isoform is a potent stimulant of Tau fibrillization. Along this line, it was previously shown that the C-terminus displays an inhibitory role in Tau assembly and that inhibition can be partly reversed by pseudo-phosphorylation at the PHF-1 epitope (S396E/S404E) or truncation of the C-terminus (Abraha et al., 2000). Moreover, we have shown in peptides that a combination of S400 *O*-GlcNAcylation and S404 phosphorylation induces similar conformational changes in the region downstream S404 as PHF1-3P. Therefore, in Tau-G/P(GSK3β), the significant amount of gS400/pS404 isoform (21%) could also contribute to a large extent to acceleration of Tau fibrillar aggregation thereby counteracting the *O*-GlcNAc-mediated inhibition. This would suggest that a combination of phosphorylation and *O*-GlcNAcylation could be deleterious in the same way than PHF-1 phosphorylation in fibrillar assembly.

We have shown here that seeds of Tau2N4R formed with heparin are able to induce fibrillar aggregation of monomeric TauS262A isoforms bearing various PTMs into PHF-like filaments as detected by TEM (Figure 6). Despite the large amount of fibrillar material, increase of ThT signals was by far weaker as compared to those of aggregation reactions of TauPHF1 series induced by heparin, and the variations of ThT emission signal is too weak to be interpreted unambiguously. It must be noticed, however, that ThT signals at the initial time is high as compared to aggregation reactions induced by heparin and higher than those of seeds alone, and there is no lag phase as well. This argues that Tau seeds promote aggregation of monomeric Tau regardless of the PTM status by reducing the nucleation rate as previously described (Meyer et al., 2014; Dinkel et al., 2015) even in the absence of external cofactors. In our setup, a 10-fold dilution of seeds made of Tau2N4R pre-incubated with heparin leads to a final heparin dilution with monomeric Tau (equivalent to 0.25 μM heparin for 25 μM monomeric Tau considering the initial heparin concentration used to prepare seeds) beyond a concentration in which heparin is able to efficiently promote aggregation (see Supplementary Figure 10C). It is likely that most of heparin used to prepare seeds is incorporated into fibrils and a very low amount is expected to be available to induce aggregation of fresh monomers. Fichou et al. have shown that Tau fibrils induced by heparin dissociate upon treatment with heparinase concluding that heparin is an essential cofactor of Tau aggregation and a cofactor is required to sustain fibril seeding (Fichou et al., 2018c, 2019). However, it appears in our study that Tau seeds without additional heparin are able to stimulate fibril formation but the content of fibrils is limited by the amount of seeds and rapidly reaches a plateau. TEM imaging of aggregation reactions of monomeric Tau and Tau-G shows the presence of individual filaments together with large clusters of fibrils while seeds alone are made of individual fibrils of shorter length (Figure 6). These observations indicate that Tau seeds allow Tau and Tau-G monomers to aggregate and *O*-GlcNAcylation does not intrinsically inhibit the fibrillization process. Phosphorylation of both Tau and Tau-G, on the other hand, induce the formation of individual fibrils to a lesser extent with greater length than seeds and no fibrillar clusters were observed indicating a distinct mechanism of fibrillization as compared to the non-phosphorylated isoforms. This suggests that at least part of heparin within Tau seeds is still accessible as shown in Fichou et al. (2018c), and reduces interactions of phosphorylated Tau species with seeds. In contrast, seeding of TauPHF1 aggregation also shows that GSK3β phosphorylation significantly stimulates the formation of PHF-like filaments of Tau, Tau-P and Tau-G isoforms as shown by a greater extent of long fibrillar species and clusters, and the significant reduction of soluble Tau monomers at aggregation end-point (see Supplementary Figures 11A,C,D).

A reciprocal regulation of Tau phosphorylation and *O*-GlcNAcylation has been shown in various cellular models of tauopathies or *in vivo* in transgenic mice. In most cases, decreasing protein *O*-GlcNAcylation through OGT knock-down or by mimicking impaired glucose metabolism and uptake as in AD brain leads to increased site-specific phosphorylation of Tau (Liu et al., 2004, 2009). Conversely, Thiamet-G, a potent OGA inhibitor, increases protein *O*-GlcNAcylation in the brain of TG mice, reduces Tau phosphorylation and alleviates Tau pathology (Yuzwa et al., 2008; Hastings et al., 2017). However, Thiamet-G injection was also shown to trigger GSK3β activation leading to an increased site-specific Tau phosphorylation (Yu et al., 2012). The proposed mechanism was that increased *O*-GlcNAcylation leads to inhibition of AKT that negatively regulates GSK3β by phosphorylation of serine 9. Here, we have shown that *O*-GlcNAcylation modulates GSK3β phosphorylation of PHF-1 epitope as previously observed in peptides (Smet-Nocca et al., 2011), but does not modify CDK2 priming nor remote phosphorylation in the proline-rich domain excluding an extended effect of *O*-GlcNAcylation on phosphorylation distributed along the entire Tau protein. Furthermore, *O*-GlcNAcylation does not antagonize the conformational changes induced by phosphorylation of S404 nor the increased fibrillization properties of GSK3β-phosphorylated Tau. Our data sustain the hypothesis that regulation of Tau phosphorylation and aggregation as well as neurodegeneration through *O*-GlcNAc modulation (e.g., with OGA inhibitors) is likely an indirect process involving the *O*-GlcNAc-mediated regulation of enzymes implicated in phosphorylation dynamics (Lubas and Hanover, 2000; Dias et al., 2012; Shi et al., 2012; Wang et al., 2012; Xie et al., 2016) or other actors in Tau pathology (Smet et al., 2004b; Landrieu et al., 2006; Lippens et al., 2007; Borghgraef et al., 2013).

## Conclusion

In this study, we have shown that GSK3β exacerbates fibrillar aggregation of Tau protein *in vitro* and significantly counteracts inhibition of fibrillization mediated by *O*-GlcNAc posttranslational modifications although *O*-GlcNAcylation severely reduces the PHF1-3P isoform. Conversely, priming by CDK2 increases GSK3β phosphorylation in both Tau and *O*-GlcNAcylated Tau isoforms by providing a number of phosphorylation sites in the proline-rich domain which otherwise is not targeted by GSK3β activity. A conformational study of the impact of PTMs in the C-terminal region around the PHF-1 epitope shows that phosphorylation of S404 triggers a dynamic conformational change in the region immediately downstream S404 site that is enhanced by other phosphorylations of the PHF-1 epitope or S400 *O*-GlcNAcylation. These data suggest that multiple phosphorylations or combination of phosphorylation and *O*-GlcNAcylation within the PHF-1 epitope have the same conformational effect while increasing the formation of PHF-like filaments.

## Data Availability Statement

The original contributions presented in the study are included in the article/Supplementary Material, further inquiries can be directed to the corresponding author.

## Author Contributions

CS-N designed research. F-XC, AL, CS-N, and XT performed research. CD, OR, NG, CH, and CS-N contributed new materials/analytic tools. F-XC, AL, XT, and CS-N analyzed data. IL and CS-N wrote the manuscript. All authors contributed to the article and approved the submitted version.

## Conflict of Interest

The authors declare that the research was conducted in the absence of any commercial or financial relationships that could be construed as a potential conflict of interest.

## Abbreviations

AD: Alzheimer’s disease
CDK2: cyclin dependent kinase 2
Fmoc: 9-fluorenylmethoxycarbonyl
HSQC: heteronuclear single quantum correlation
IPTG: isopropyl- β-D -1-thiogalactopyranoside
MALDI-TOF MS: matrix-assisted laser desorption ionization-time-of-flight mass spectrometry
NOESY: Nuclear Overhauser SpectroscopY
pS: phosphorylated Ser
gS: O-GlcNAcylated Ser
GSK3β: glycogen synthase kinase 3 β
MT: microtubule
PTM: posttranslational modification
*O*-GlcNAc: *O*- β-linkedN-acetylglucosamine
OGT: *O*-GlcNAc transferase
SDS-PAGE: sodium dodecyl sulfate-polyacrylamide gel electrophoresis
PHF: paired helical filaments
RP-HPLC: reverse phase high performance liquid chromatography
TFA: trifluoroacetic acid
THP: Tris(hydroxypropyl)phosphine
TOCSY: Total Correlation SpectroscopY.
