# Supplementary material for: Phosphorylation and O-GlcNAcylation of the PHF-1 Epitope of Tau Protein Induce Local Conformational Changes of the C-Terminus and Modulate Tau Self-Assembly Into Fibrillar Aggregates
Source: Front Mol Neurosci. 2021 Jun 17;14:661368. doi: 10.3389/fnmol.2021.661368 (PMC8249575; doi:10.3389/fnmol.2021.661368)
Supplement: Supplementary file 1 [file Data_Sheet_1.docx]

**Supplementary Information**

Table S1

| TauPHF1-P(GSK3) | |  |  |  |
| --- | --- | --- | --- | --- |
| **Assignment** | **δ(1H) (ppm)** | **δ(15N) (ppm)** | **Intensity** | **Integral** |
| S396 | 8.3582 | 119.3682 | 1.07E+10 | 2.47E+11 |
| S404 | 8.3582 | 120.0031 | 1.48E+10 | 3.00E+11 |
| S400 | 8.4722 | 120.174 | 1.37E+10 | 3.25E+11 |
| pS404(pS400) | 8.6171 | 120.0763 | 2.72E+10 | 5.10E+11 |
| pS396 | 8.8667 | 121.7611 | 2.08E+10 | 3.91E+11 |
| pS400(pS404) | 8.9437 | 121.3216 | 2.36E+10 | 4.64E+11 |
|  |  |  |  |  |
| TauPHF1-G |  |  |  |  |
| **Assignment** | **δ(1H) (ppm)** | **δ(15N) (ppm)** | **Intensity** | **Integral** |
| (gS412)S413 | 8.3073 | 117.9241 | 3.94E+10 | 5.67E+11 |
| S404(S400) | 8.3474 | 119.9507 | 5.27E+10 | 5.33E+11 |
| S396 | 8.3567 | 119.3647 | 1.17E+11 | 1.39E+12 |
| S404(gS400) | 8.3659 | 120.0118 | 9.05E+10 | 9.21E+11 |
| gS412 | 8.4167 | 119.0229 | 3.48E+10 | 4.69E+11 |
| gS400 | 8.4167 | 119.4624 | 6.91E+10 | 8.58E+11 |
| S412(gS413) | 8.4399 | 119.3403 | 5.89E+10 | 8.34E+11 |
| S412(S413) | 8.4691 | 119.499 | 3.84E+10 | 4.99E+11 |
| S400 | 8.4707 | 120.1705 | 4.75E+10 | 6.18E+11 |
| (S412)gS413 | 8.5092 | 117.9608 | 6.68E+10 | 7.22E+11 |
| (S412)S413 | 8.5169 | 118.3636 | 4.27E+10 | 5.74E+11 |
|  |  |  |  |  |
| TauPHF1-G/P(GSK3) | |  |  |  |
| **Assignment** | **δ(1H) (ppm)** | **δ(15N) (ppm)** | **Intensity** | **Integral** |
| (gS412)S413 | 8.3228 | 117.979 | 2.71E+09 | 7.61E+10 |
| S404(S400) | 8.3644 | 120.0056 | 3.33E+09 | 5.06E+10 |
| S396(pS400) | 8.366 | 119.3829 | 1.01E+10 | 1.93E+11 |
| S396(S400) | 8.3798 | 119.4928 | 2.52E+09 | 8.34E+10 |
| S404(gS400) | 8.3829 | 120.0666 | 9.71E+09 | 1.79E+11 |
| gS400 | 8.4245 | 119.505 | 1.02E+10 | 2.52E+11 |
| gS412 | 8.4291 | 119.0411 | 4.40E+09 | 1.04E+11 |
| S412(gS413) | 8.443 | 119.3097 | 7.02E+09 | 1.71E+11 |
| S412(S413) | 8.4738 | 119.5416 | 3.44E+09 | 1.13E+11 |
| S400 | 8.4831 | 120.2009 | 3.07E+09 | 1.02E+11 |
| (S412)gS413 | 8.5077 | 117.9912 | 2.87E+09 | 1.19E+11 |
| (S412)S413 | 8.5231 | 118.394 | 1.72E+09 | 5.94E+10 |
| pS404(pS400) | 8.6264 | 120.0666 | 2.84E+09 | 5.76E+10 |
| pS404(S400) | 8.6449 | 120.2986 | 7.41E+08 | 1.32E+10 |
| pS404(gS400) | 8.6865 | 120.3474 | 4.41E+09 | 8.00E+10 |
| pS396 | 8.8822 | 121.8246 | 2.57E+09 | 4.57E+10 |
| pS400 | 8.9577 | 121.3607 | 2.77E+09 | 5.62E+10 |

Table S1: ^1^H and ^15^N chemical shifts, peak intensity and peak integration of phosphorylated and/or O-GlcNAcylated residues in TauPHF1-P(GSK3), TauPHF1-G and TauPHF1-G/P(GSK3).

Table S2

| TauPHF1-P(GSK3) | |  |  |  |
| --- | --- | --- | --- | --- |
| **% phosphorylation** | **intensity** | **integral** |  |  |
| pS396 | 66.1% | 61.3% |  |  |
| pS400 | 63.3% | 58.8% |  |  |
| pS404 | 64.7% | 63.0% |  |  |
|  |  |  |  |  |
| TauPHF1-G |  |  |  |  |
| **% O-GlcNAcylation** | **intensity** | **integral** |  |  |
| gS400 | 59.3% | 58.1% |  |  |
| gS412 | 26.4% | 26.0% |  |  |
| gS413 | 44.8% | 38.8% |  |  |
|  |  |  |  |  |
| TauPHF1-G/P(GSK3) | |  |  |  |
|  | ***intensity*** | ***integral*** | ***intensity*** | ***integral*** |
|  | **% phosphorylation** | | **% O-GlcNAcylation** | |
| S396 | 16.9% | 14.2% |  |  |
| S400 | 17.2% | 13.7% | 63.6% | 61.4% |
| S404 | 38.0% | 39.7% |  |  |
| S412 |  |  | 29.6% | 26.8% |
| S413 |  |  | 39.3% | 46.8% |

Table S2: Phosphorylation and/or *O*-GlcNAcylation levels measured either with peak intensities or peak integrations in TauPHF1-P(GSK3), TauPHF1-G and TauPHF1-G/P(GSK3).

Figure S1


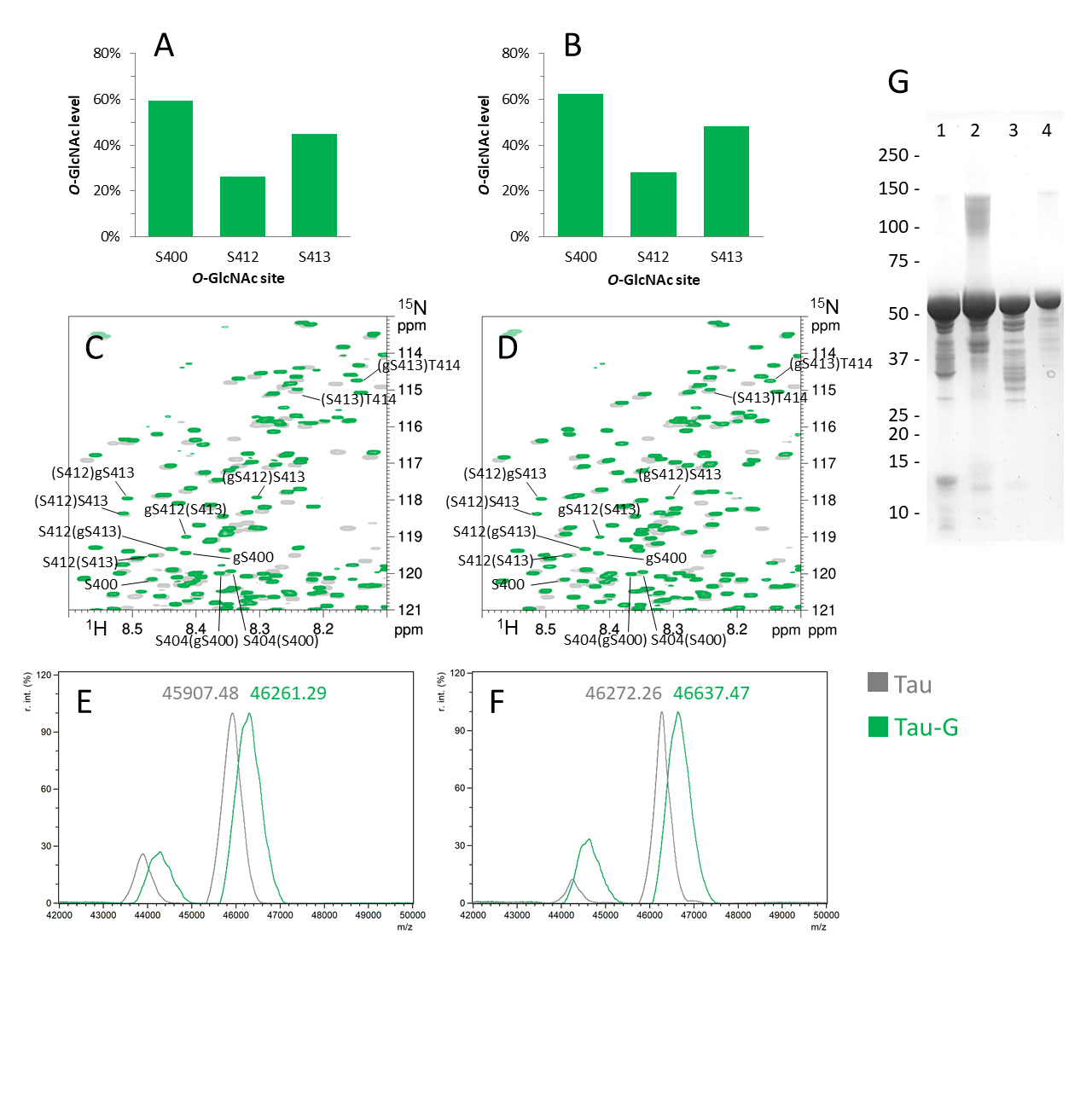


**Figure S1:** *O*-GlcNAcylation of TauS262A and TauPHF1 by recombinant OGT. (A,B) Quantitative *O*-GlcNAc patterns of TauPHF1 (A) and TauS262A (B) determined by using NMR ^1^H-^15^N HSQC spectra of TauPHF1-G (C, green) and TauS262A-G (D, green), respectively. ^1^H-^15^N HSQC spectra of non-glycosylated TauPHF1 (C, grey) and TauS262A (D, grey) are indicated as a control. (E,F) Mass spectrometry analyses of TauPHF1 (E) and TauS262A (F) in their non glycosylated (grey) and *O*-GlcNAcylated (green) forms indicating a m/z increment of +353 and +365 corresponding to the addition of 1.7 and 1.8 GlcNAc per tau, respectively. (G) SDS-PAGE analyses of TauS262A (lane 1), TauS262A-G (lane 2), TauPHF1 (lane 3) and TauPHF1-G (lane 4).

Figure S2


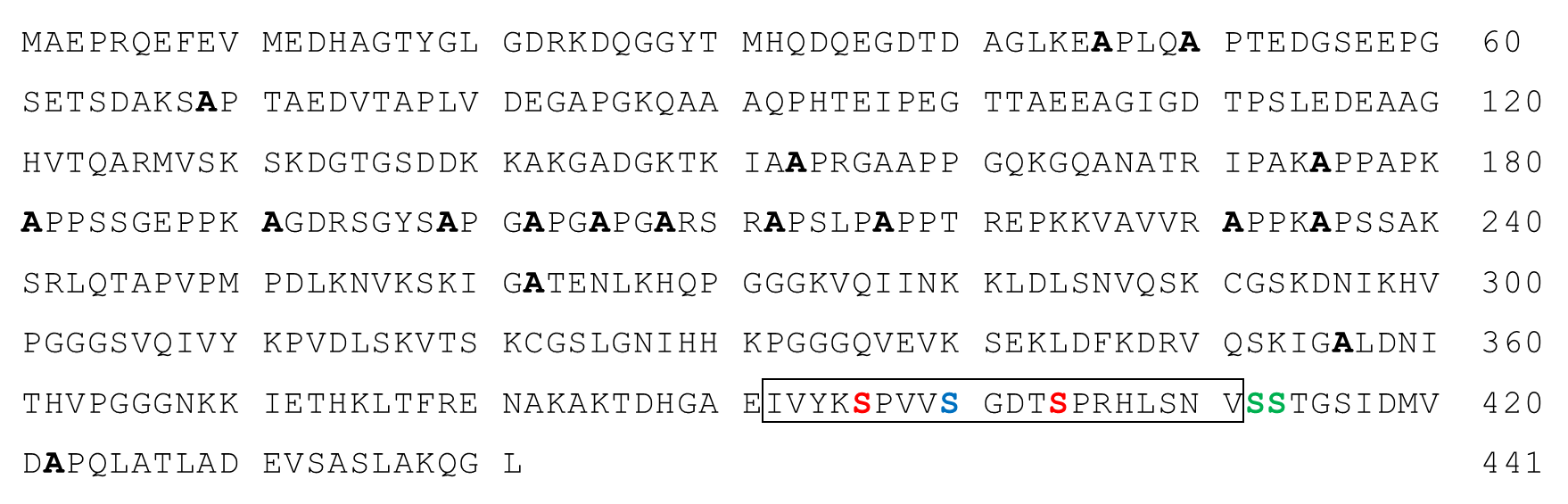


**Figure S2:** Sequence of Tau PHF1 mutant. Ser/Thr residues mutated into Ala are annotated in bold while remaining Ser-Pro sites (S396 and S404) within the PHF-1 epitope are indicated in red. *O*-GlcNAc sites obtained using recombinant ncOGT glycosylation reaction are indicated in green (S412, S413). S400 residue that is either phosphorylated or *O*-GlcNAcylated is indicated in blue. Boxed sequence corresponds to Tau[392-411] peptide.

Figure S3


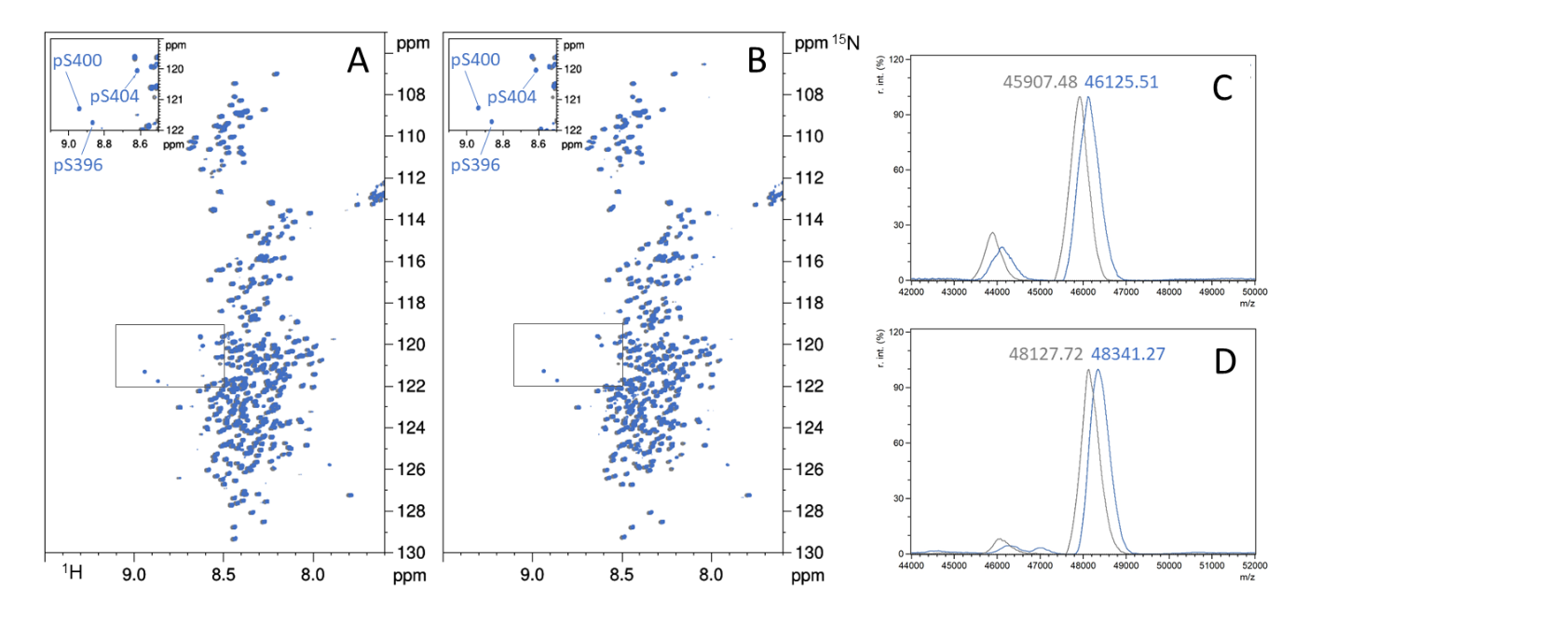


**Figure S3:** Phosphorylation of TauPHF1 and TauS262A by GSK3β without CDK2 priming. ^1^H-^15^N HSQC spectra (A,B) and mass spectra (C,D) of ^15^N-TauPHF1 (A,C) and ^15^N^13^C-TauS262A (B,D) before (grey) and after GSK3β phosphorylation (blue) indicate the same pattern of phosphorylation targeting only the PHF-1 phospho-epitope. Resonances of phospho-residues (boxed regions in A and B) are depicted in the inset.

Figure S4


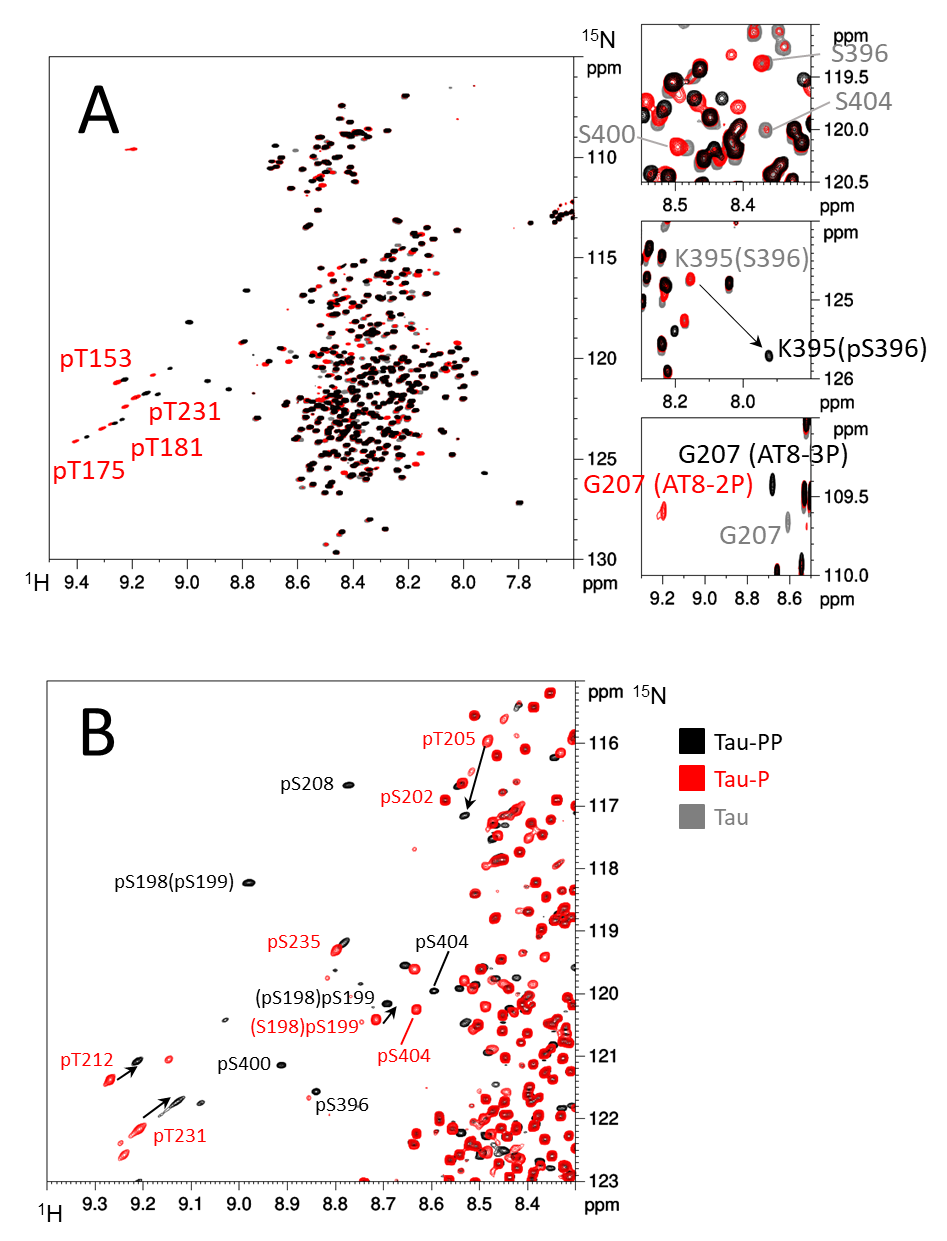


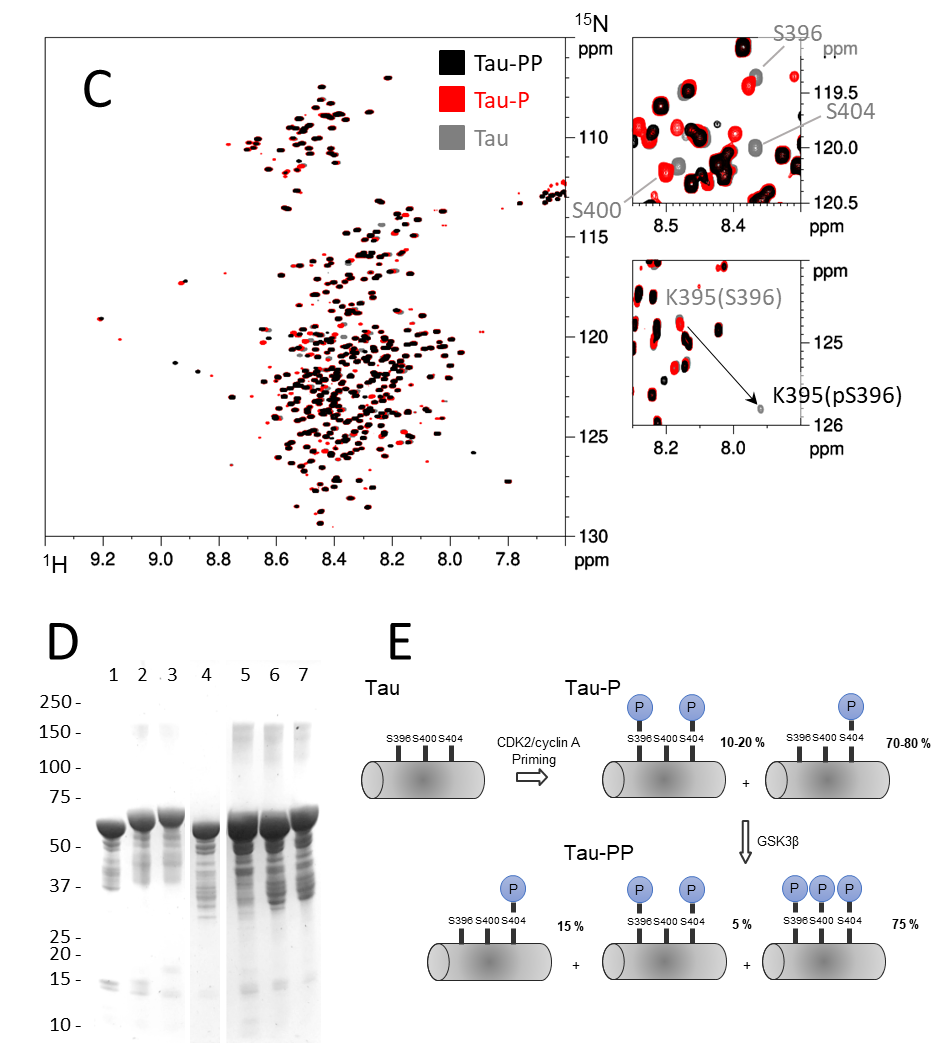


**Figure S4:** Phosphorylation of TauPHF1 and TauS262A. (A-C) NMR ^1^H-^15^N HSQC spectra of ^15^N-TauS262A (A,B) and ^15^N-TauPHF1 (C) in their non-phosphorylated (grey), CDK2-phosphorylated (red) and CDK2/GSK3β-phosphorylated (black) forms. A zoom of resonances of TauS262A phosphorylation sites is depicted in (B). (D) SDS-PAGE analysis of phosphorylation of TauS262A (lanes 1-3) and TauPHF1 (lanes 4-7): non-phosphorylated Tau (lanes 1, 4), CDK2-phosphorylated Tau (lanes 2, 6), CDK2/GSK3β-phosphorylated Tau (lanes 3, 7) and GSK3β-phosphorylated Tau (lane 5). (E) Phosphorylation patterns of the PHF-1 epitope within TauPHF1 and TauS262A proteins showing site-specific phosphorylation levels after CDK2 priming and sequential CDK2/GSK3β phosphorylation as well as distribution of the major phospho-isoforms. The remaining isoforms of PHF-1 epitope in Tau-P and Tau-PP representing 10% and 5% of the population, respectively, that cannot be unambiguously defined (e.g the remaining isoforms could be either non-phosphorylated or phosphorylated on S396 or a mix of both) is not depicted.

Figure S5

A


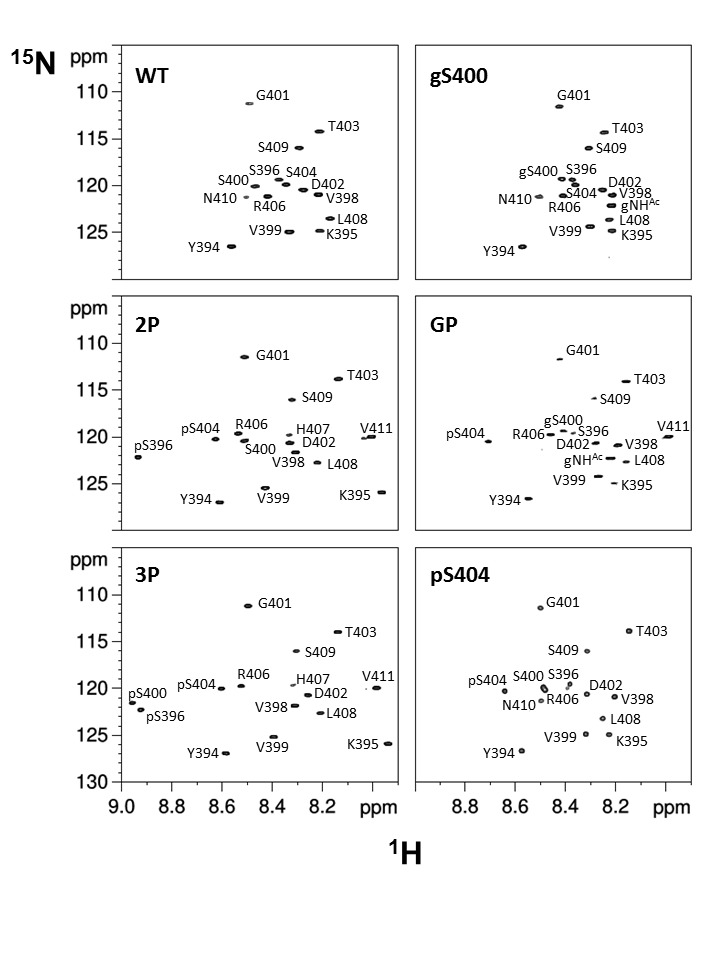


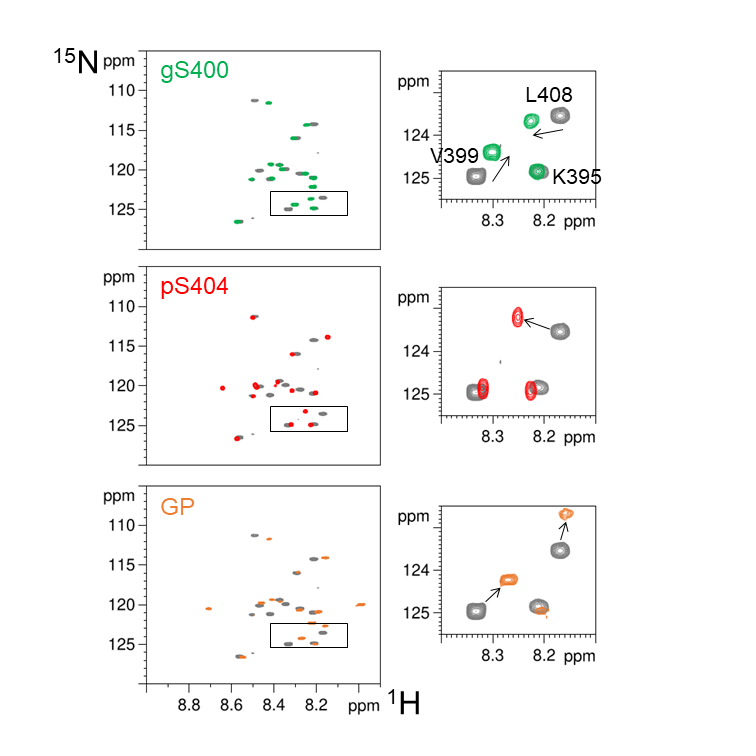


B

**Figure S5:** (A) ^1^H-^15^N HSQC spectra of Tau[392-411] peptide series with various PTMs (see Table 2). WT indicates peptide without PTM. gNH^Ac^ corresponds to the N-acetamide group of GlcNAc. (B) ^1^H-^15^N HSQC spectra correspond to the Tau[392-411] peptide without PTM (grey), S400 *O*-GlcNAcylated peptide (gS400, green), S404 phosphorylated peptide (pS404, red) and S400 *O*-GlcNAcylated, S404 phosphorylated peptide (GP, orange). Zoomed region of spectra on the right shows the amide resonances of L408 that is shifted upon distant S404 phosphorylation or S400 *O*-GlcNAcylation or both modifications in GP illustrating long-range effect of PTMs in the C-terminal region of peptides.

Figure S6


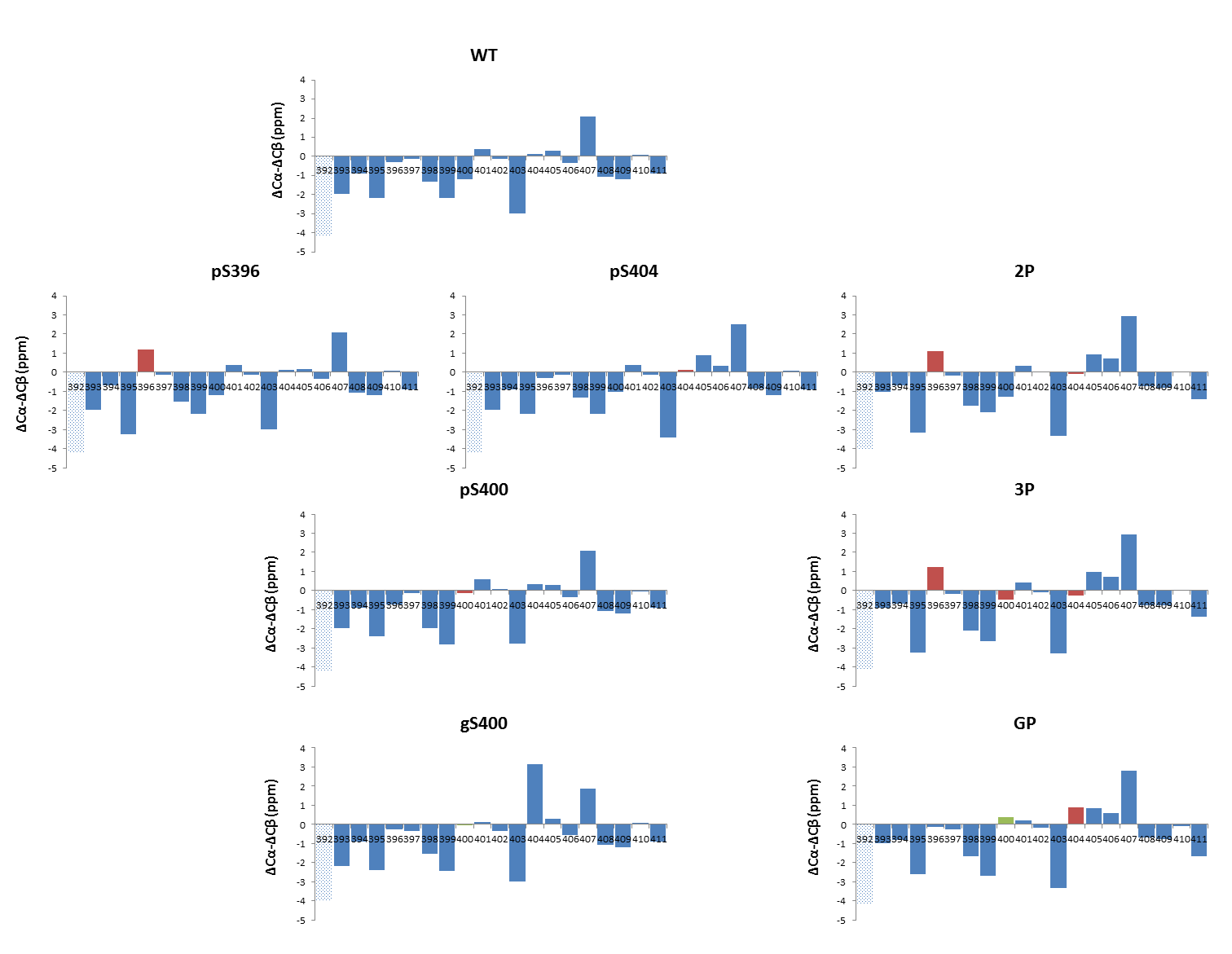


A

**
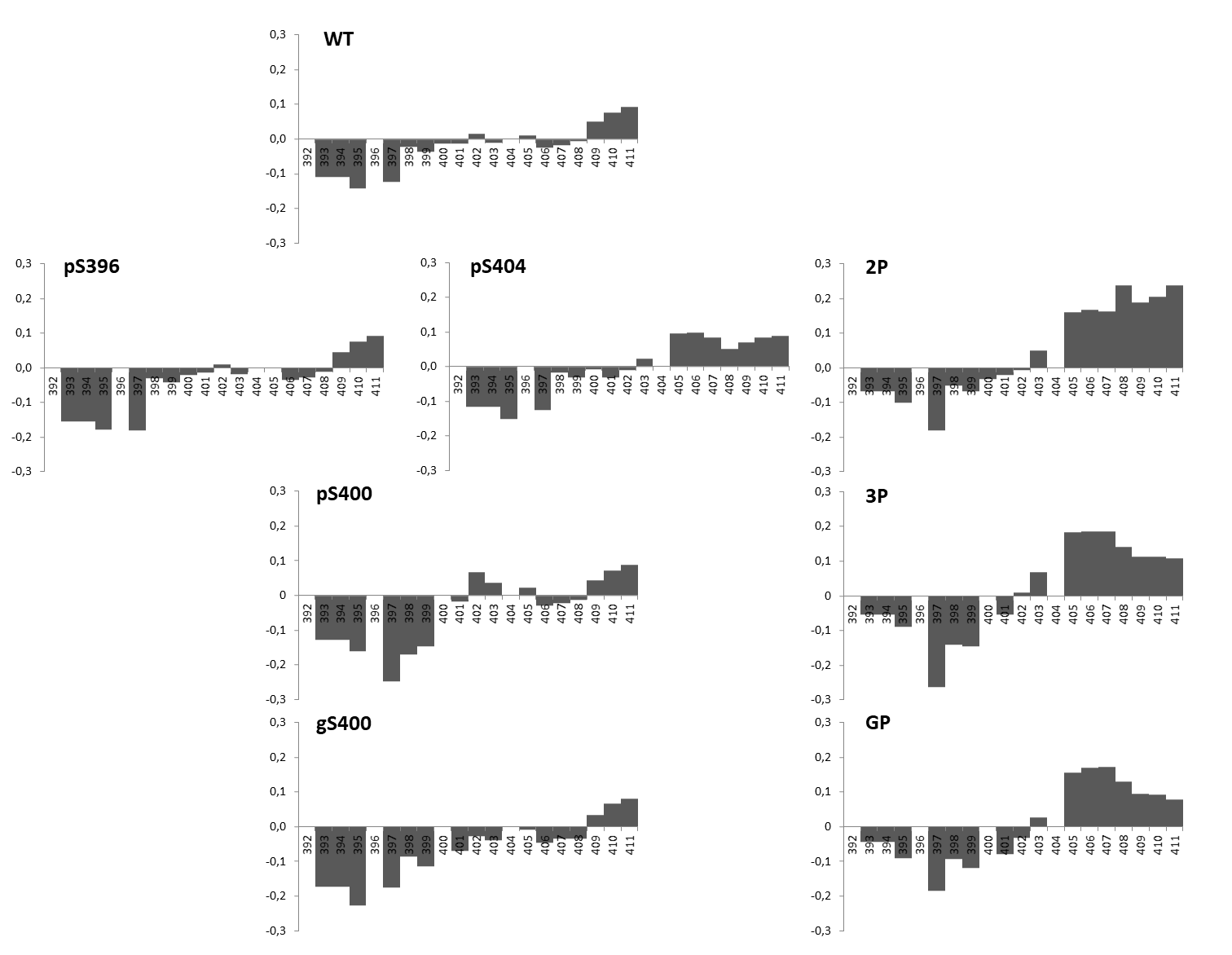
**

B

**Figure S6 :** (A,B) NMR chemical shift analyses of Tau[392-411] peptide series with various PTMs (listed in Table 2) for their conformational propensity. Peptide without PTM is indicated as “WT”. (A) Secondary chemical shifts Δ(Cα-Cβ) of Tau[392-411] peptides. Phosphorylated Ser are indicated by red bars and *O*-GlcNAcylated Ser by green bars. For *O*-GlcNAc Ser, random coil values of 56.5 ppm and 70.7 ppm for Cα and Cβ, respectively, were used based on previous identifications of *O*-GlcNAc sites in random coil peptides and proteins (1–4). (B) SSP scores of Tau[392-411] peptides using ^1^Hα, ^13^Cα and ^13^Cβ chemical shifts.

Figure S7

A

B


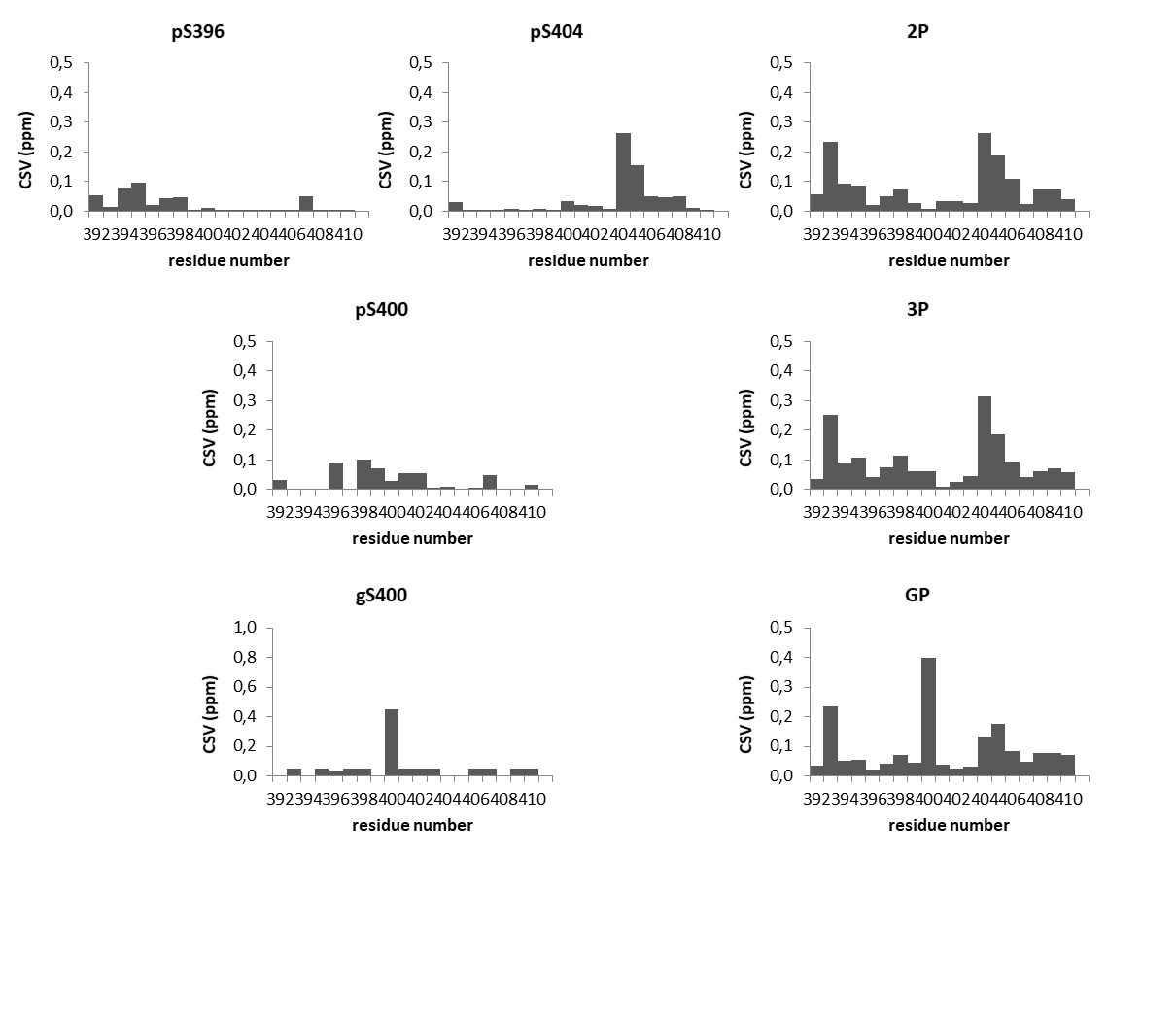


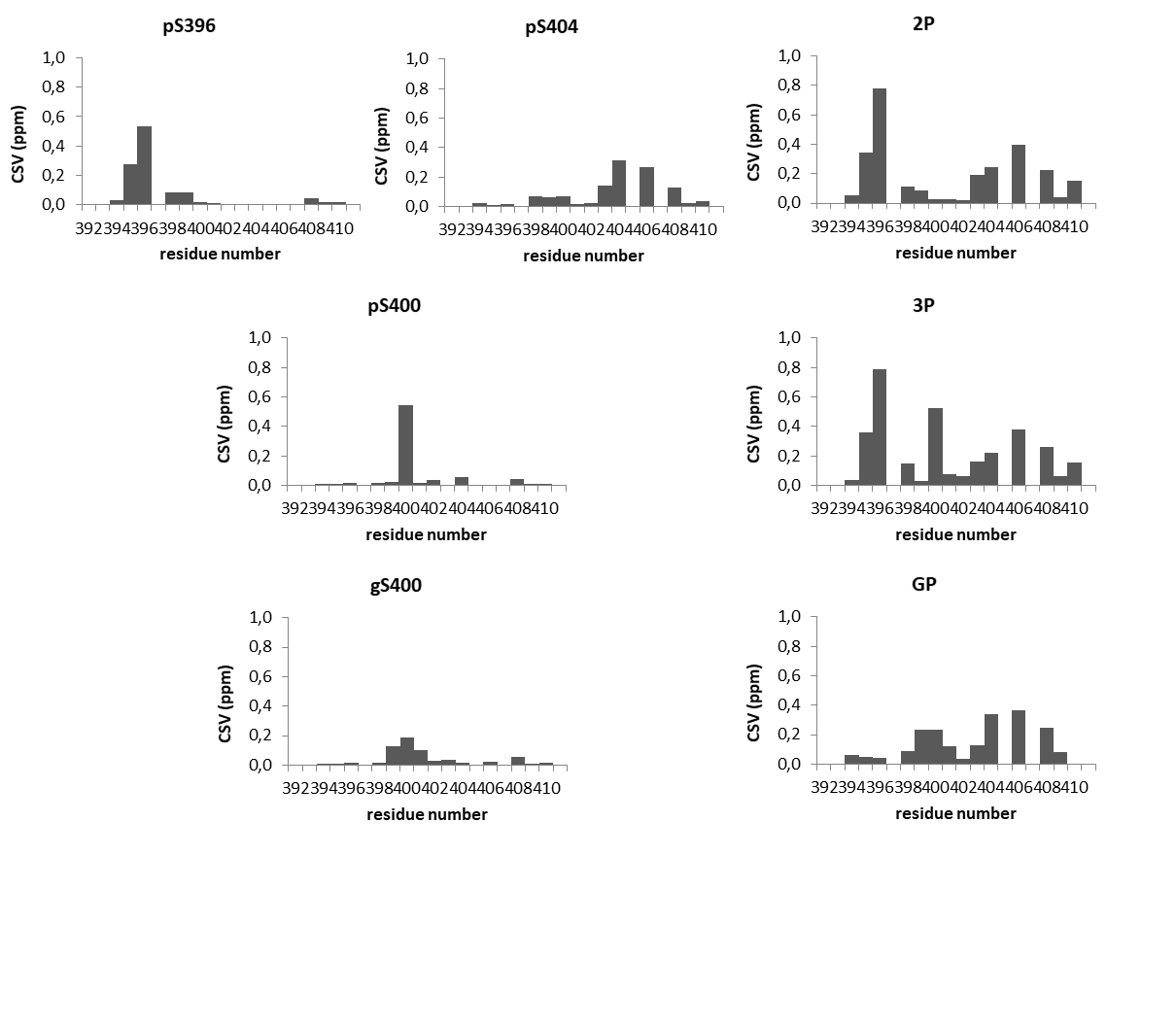


**Figure S7 :** Chemical shift variations of Hα/Cα (A) and Hn/N (B) of Tau[392-411] peptide series with various PTMs (see Table 2) as compared to the peptide without PTM.

Figure S8


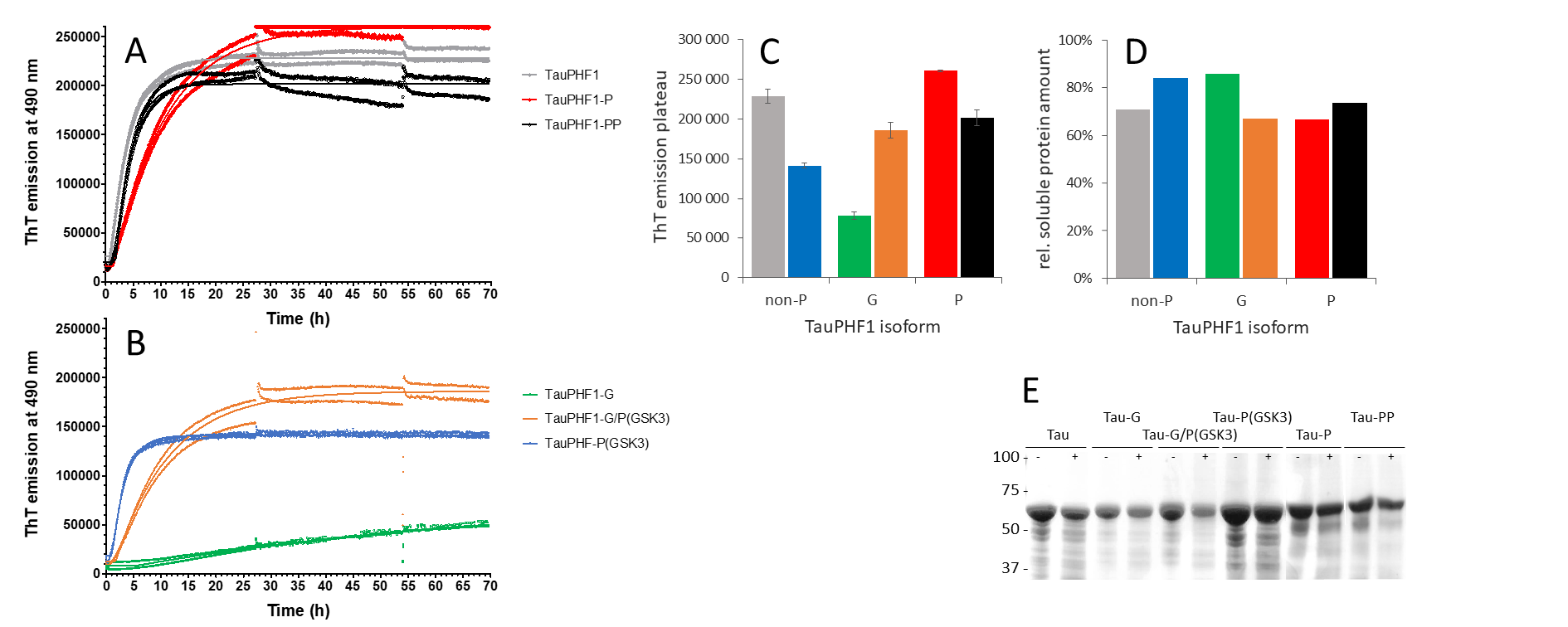


**Figure S8:** Aggregation of TauPHF1 isoforms induced by heparin. (A,B) Time-course of TauPHF1 fibrillar aggregation induced by heparin (at a Tau:heparin ratio of 4:1) over 70 hours at 37°C as detected by ThT fluorescence at 490 nm. Tau protein without PTM is depicted in grey, Tau-P in red, Tau-PP in black, Tau-P(GSK3β) in blue, Tau-G in green and Tau-G/P(GSK3β) in orange. Aggregation reactions were performed in duplicate that are plotted for each protein as well as the fitted one-phase association curve. (C) Graphical representation of ThT plateau for the different TauPHF1 isoforms at end-point of aggregation. (D,E) Difference in soluble protein amount at t=0 (-) and after 70 hours (+) of aggregation with heparin measured by integration of bands in SDS-PAGE at 4%-20% polyacrylamide (E). (D) Graphical representation of ratios of soluble protein amount at the end-point of aggregation on the initial amount. Bars in (C,D) graphics are depicted with the same color coding as in (A,B).

Figure S9


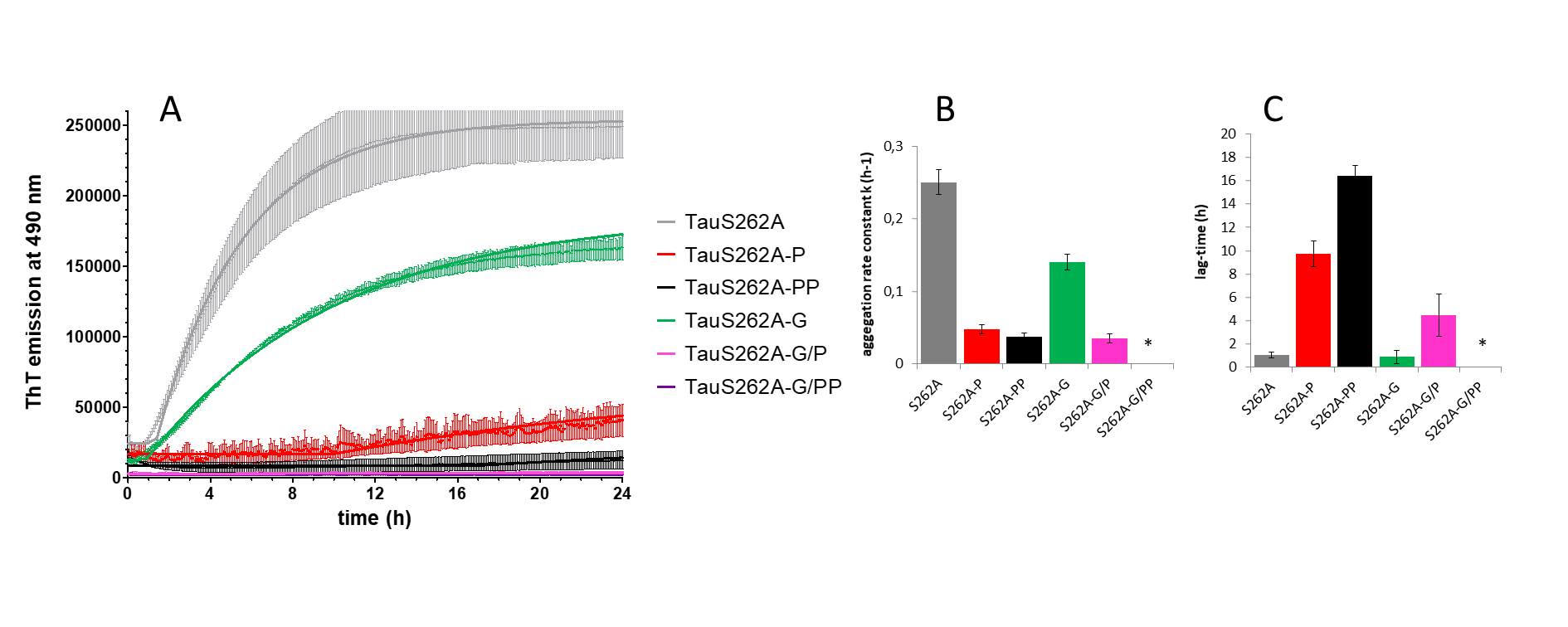


**Figure S9:** (A) Time course of TauS262A fibrillar aggregation induced by heparin (Tau:heparin ratio of 4:1) at 37°C as detected by ThT fluorescence at 490 nm for Tau protein without PTM (grey), Tau-P (red), Tau-PP (black), Tau-G (green), Tau-G/P (pink) and Tau-G/PP (purple). Aggregation reactions were performed in at least duplicate for each protein. Data are plotted as mean ± SD together with fitted one-phase association curves. (B,C) Graphical representation of elongation rate constants k (B) and lag-times (C) with the same color coding as in (A). Asterisks indicate data that cannot be fitted due to very low aggregation rate.

Figure S10

**
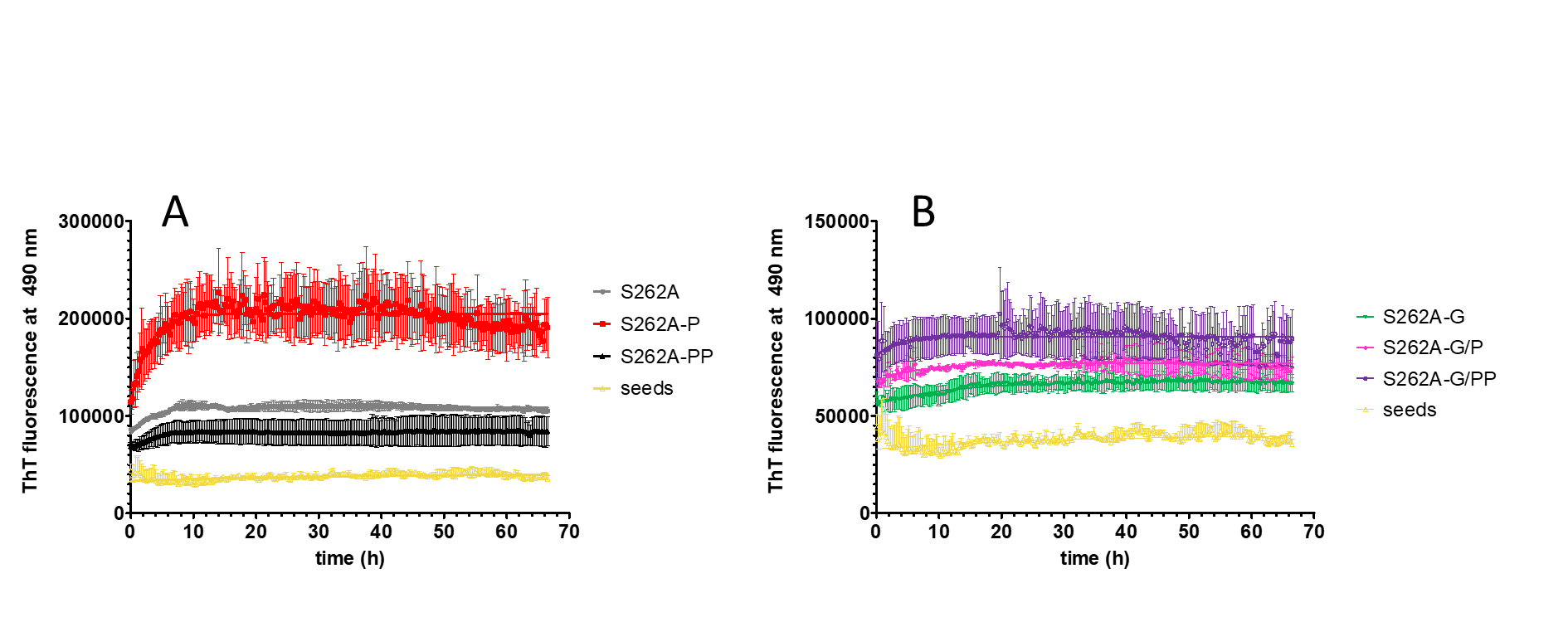

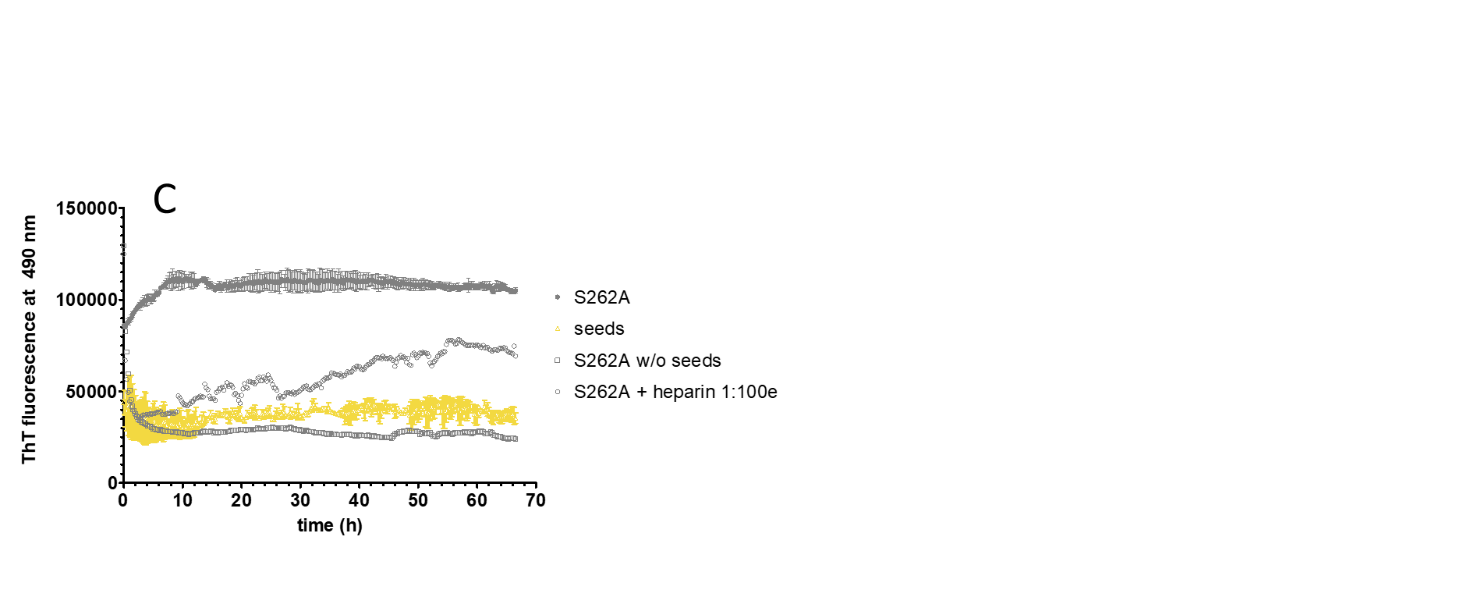
**

**Figure S10:** (A,B) Aggregation reactions of TauS262A series at 25µM monomeric Tau with seeds made with TauS262A incubated with heparin (10 µM Tau : 2.5 µM heparin). Aggregation of Tau (grey), Tau-P (red), Tau-PP (black), Tau-G (green), Tau-G/P (pink), Tau-G/PP (purple). Reactions were performed in duplicate and data were plotted as mean ± SD. Aggregation reaction of seeds without monomeric Tau is indicated by open yellow triangles (A-C). (C) Control of aggregation reactions of TauS262A without seeds (open grey squares) or with the same heparin concentration than seeds dilution (0.25 µM for 25 µM of TauS262A) (open grey circles). Aggregation of TauS262A with seeds (plain grey circles) or seeds without monomeric Tau (yellow) that are the same as in (A) are shown for comparison.

Figure S11


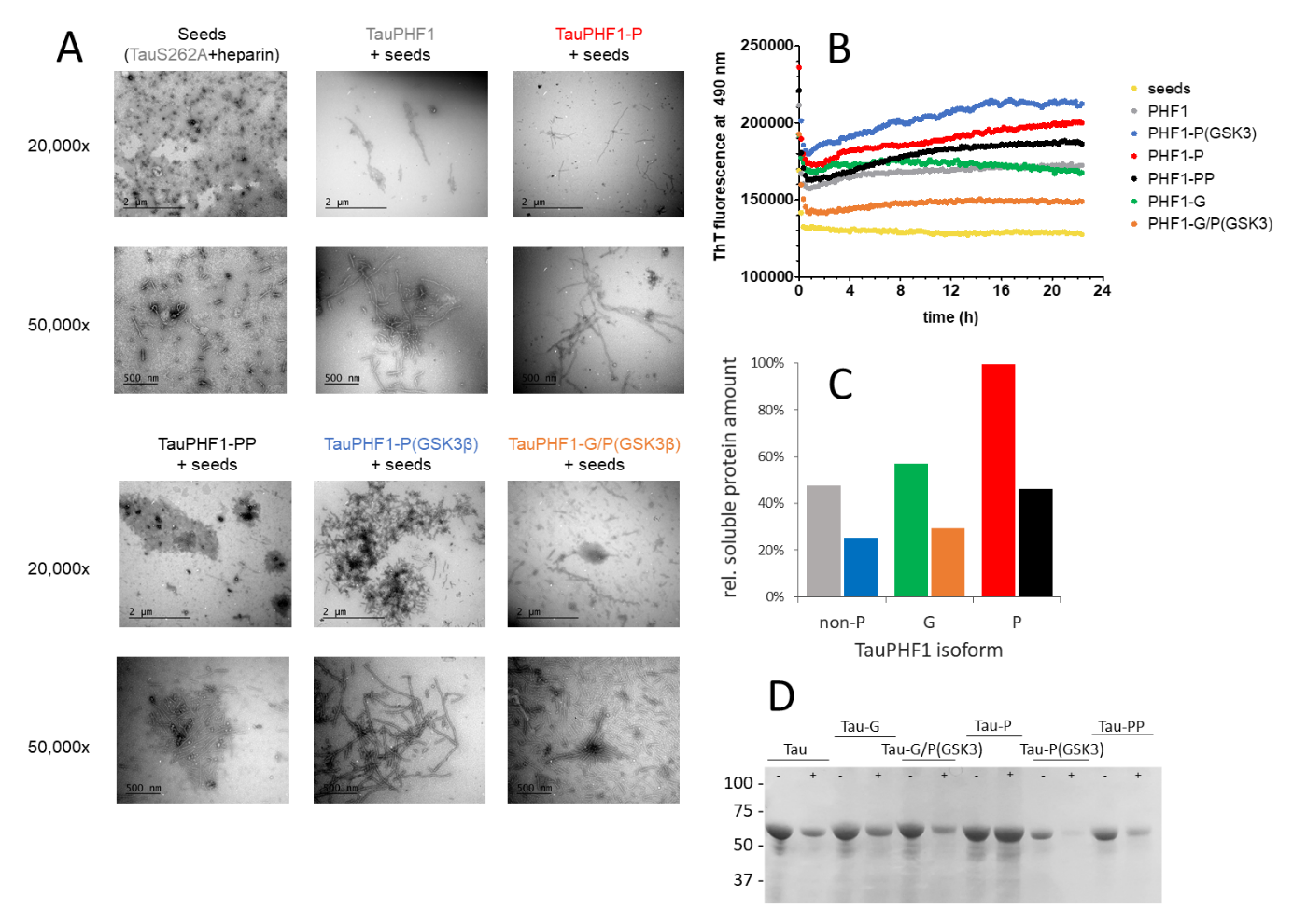


**Figure S11:** Aggregation of TauPHF1 isoforms induced by seeds. (A) Transmission electron micrographs with negative staining of fibrillar aggregation of TauPHF1 protein series induced by Tau seeds prepared with TauS262A and heparin, diluted 10-fold in 25 µM monomeric Tau (grey), Tau-P (red), Tau-PP (black), Tau-P(GSK3β) (blue) and Tau-G/P(GSK3β) (orange) showing two different magnifications: 20,000X (upper panels) and 50,000X (lower panels). The scale bar of 2 µm applies to upper panels, 500 nm to lower panels. (B) Aggregation reactions followed by fluorescence of ThT. (C,D) Difference in soluble protein amount at t=0 (-) and after 70 hours (+) of aggregation with seeds measured by integration of bands in SDS-PAGE at 4%-20% polyacrylamide(D). (C) Graphical representation of ratios of soluble protein amount at the end-point of aggregation on the initial amount. Bars are depicted with the same color coding as in (A,B).

Figure S12


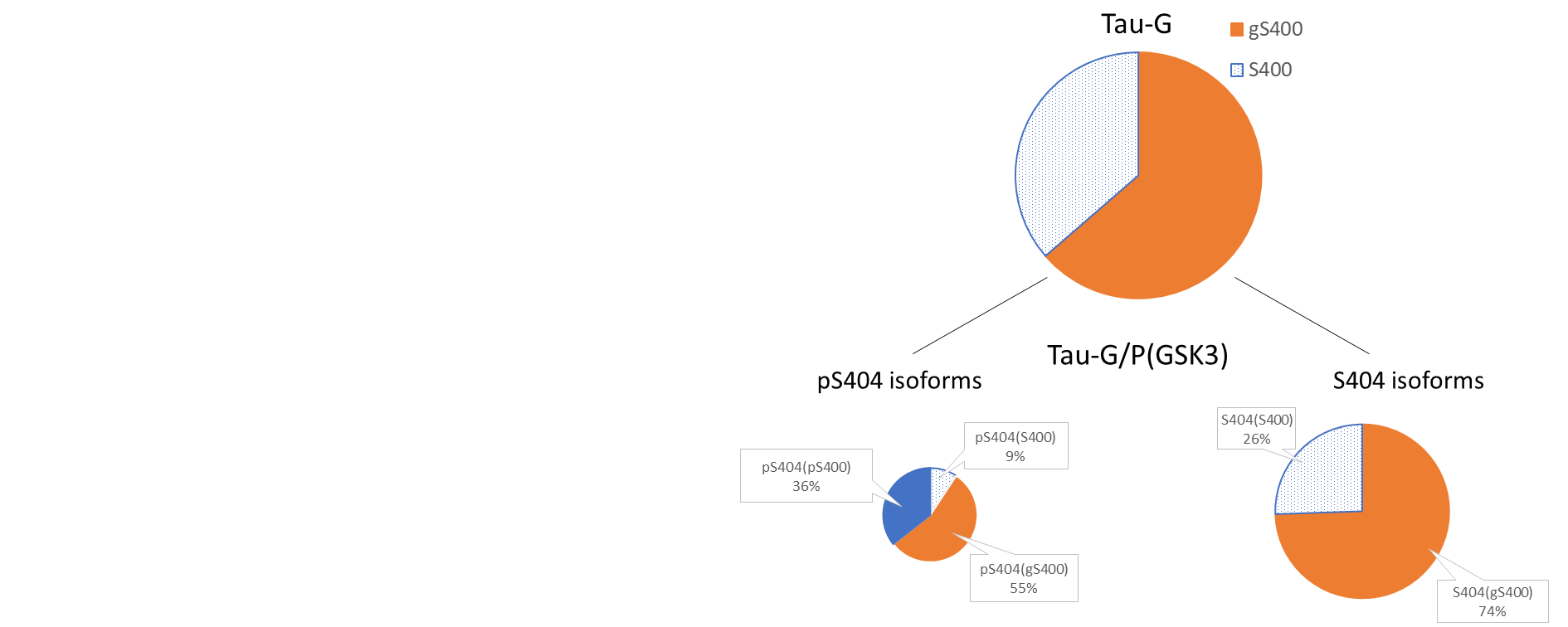


**Figure S12:** Distribution of S400 O-GlcNAcylation forms in S404 and pS404 isoforms upon GSK3β phosphorylation of Tau-G. S400 *O*-GlcNAcylation level of 60% in Tau-G (orange section, upper chart) is distributed differentially in pS404 and S404 isoforms resulting from GSK3β phosphorylation into 55% and 74%, respectively (orange sections, lower charts). Non-glycosylated isoforms are indicated by blue sections, either in dotted sections for non-phosphorylated S400 or filled section for pS400.

**References**

1. Smet-Nocca C, Broncel M, Wieruszeski JM, Tokarski C, Hanoulle X, Leroy A, Landrieu I, Rolando C, Lippens G, Hackenberger CP. Identification of O-GlcNAc sites within peptides of the Tau protein and their impact on phosphorylation. *Mol Biosyst* (2011) **7**:1420–9. doi:10.1039/c0mb00337a

2. Bourré G, Cantrelle F-X, Kamah A, Chambraud B, Landrieu I, Smet-Nocca C. Direct Crosstalk Between O-GlcNAcylation and Phosphorylation of Tau Protein Investigated by NMR Spectroscopy. *Frontiers in Endocrinology* (2018) **9**: doi:10.3389/fendo.2018.00595

3. Dubois-Deruy E, Belliard A, Mulder P, Bouvet M, Smet-Nocca C, Janel S, Lafont F, Beseme O, Amouyel P, Richard V, et al. Interplay between troponin T phosphorylation and O-N-acetylglucosaminylation in ischaemic heart failure. *Cardiovasc Res* (2015) **107**:56–65. doi:10.1093/cvr/cvv136

4. Smet-Nocca C, Page A, Cantrelle F-X, Nikolakaki E, Landrieu I, Giannakouros T. The O-β-linked N-acetylglucosaminylation of the Lamin B receptor and its impact on DNA binding and phosphorylation. *Biochim Biophys Acta* (2018) **1862**:825–835. doi:10.1016/j.bbagen.2018.01.007
